# Alterations in the gut bacterial microbiome in fungal Keratitis patients

**DOI:** 10.1371/journal.pone.0199640

**Published:** 2018-06-22

**Authors:** Sama Kalyana Chakravarthy, Rajagopalaboopathi Jayasudha, Konduri Ranjith, Anirban Dutta, Nishal Kumar Pinna, Sharmila S. Mande, Savitri Sharma, Prashant Garg, Somasheila I. Murthy, Sisinthy Shivaji

**Affiliations:** 1 Jhaveri Microbiology Centre, Brien Holden Eye Research Centre, L. V. Prasad Eye Institute, Kallam Anji Reddy campus, Hyderabad, India; 2 Bio-Sciences R&D Division, TCS Research, Tata Consultancy Services Ltd., Pune, India; 3 Tej Kohli Cornea Institute, L. V. Prasad Eye Institute, Kallam Anji Reddy campus, Hyderabad, India; University of Hyderabad, INDIA

## Abstract

Dysbiosis in the gut microbiome has been implicated in several diseases including auto-immune diseases, inflammatory diseases, cancers and mental disorders. Keratitis is an inflammatory disease of the eye significantly contributing to corneal blindness in the developing world. It would be worthwhile to investigate the possibility of dysbiosis in the gut microbiome being associated with Keratitis. Here, we have analyzed fungal and bacterial populations in stool samples through high-throughput sequencing of the ITS2 region for fungi and V3-V4 region of 16S rRNA gene for bacteria in healthy controls (HC, n = 31) and patients with fungal keratitis (FK, n = 32). *Candida albicans* (2 OTUs), *Aspergillus* (1 OTU) and 3 other denovo-OTUs were enriched in FK samples and an unclassified denovo-OTU was enriched in HC samples. However, the overall abundances of these ‘discriminatory’ OTUs were very low (< 0.001%) and not indicative of significant dysbiosis in the fungal community inhabiting the gut of FK patients. In contrast, the gut bacterial richness and diversity in FK patients was significantly decreased when compared to HC. 52 OTUs were significantly enriched in HC samples whereas only 5 OTUs in FK. The OTUs prominently enriched in HC were identified as *Faecalibacterium prausnitzii*, *Bifidobacterium adolescentis*, *Lachnospira*, *Mitsuokella multacida*, *Bacteroides plebeius*, *Megasphaera* and Lachnospiraceae. In FK samples, 5 OTUs affiliated to *Bacteroides fragilis*, *Dorea*, *Treponema*, Fusobacteriaceae, and Acidimicrobiales were significantly higher in abundance. The functional implications are that *Faecalibacterium prausnitzii*, an anti-inflammatory bacterium and *Megasphaera*, *Mitsuokella multacida* and *Lachnospira* are butyrate producers, which were enriched in HC patients, whereas *Treponema* and *Bacteroides fragilis*, which are pathogenic were abundant in FK patients, playing a potential pro-inflammatory role. Heatmap, PCoA plots and functional profiles further confirm the distinct patterns of gut bacterial composition in FK and HC samples. Our study demonstrates dysbiosis in the gut bacterial microbiomes of FK patients compared to HC. Further, based on inferred functions, it appears that dysbiosis in the gut of FK subjects is strongly associated with the disease phenotype with decrease in abundance of beneficial bacteria and increase in abundance of pro-inflammatory and pathogenic bacteria.

## Introduction

Microbiome refers to the multi-species community of microorganisms (bacteria, fungi and viruses) that colonizes the surface of the body [1]. In the adult human being, the gut microbiome has about 10^14^ bacteria and together they have 200 to 300 times more genes than in humans. The abundance and diversity of the gut microbiota is distinct from the microbiota associated with other parts of the body (skin, oral cavity, specific parts of the digestive tract, vagina etc.) [2–4]. Various factors such as age, sex, dietary composition, genetic variations and pathological state also influence the composition and abundance of the gut microbiome [5–8], thus becoming relevant to human welfare [9, 10]. The predominant gut bacteria in healthy individuals are affiliated to the phyla, Bacteroidetes and Firmicutes (together 70–90%) and it also includes Actinobacteria and Proteobacteria. These four phyla constitute the core microbiome of the human gut. The core microbiome is a reflection of the healthy state of the individual and this is emphasized by the observations that in individuals with several diseases such as immune diseases, inflammatory diseases, cancers and mental disorders [11–15], the core microbiome is altered and this change is referred to as “dysbiosis”. The specific mechanism as to how dysbiosis influences human health or disease is not clearly understood. But, the available studies do indicate a pro-active role for the gut microbiome in modulating the immune system through the production of biomolecules with immunogenic activity [16, 17].

Implicating dysbiosis of gut microbiome in digestive tract diseases / diet related diseases (obesity, inflammatory bowel disease, enterocolitis, diabetes etc.) may be expected. But, when gut dysbiosis is implicated in extra-intestinal diseases like cancers, muscular dystrophy, mental disorders, vaginosis etc. it is all the more challenging [12–14]. An additional challenge would be to ascertain the role of gut microbiome in ocular diseases, which are as remote as the brain. Recent studies both in mice and human beings have clearly demonstrated an association of gut microbiome dysbiosis with Uveitis, age-related macular degeneration (AMD) and Sjogrens syndrome-associated dry eye [18–24]. The present study is an attempt to explore the possibility that dysbiosis of the gut microbiome may be associated with Keratitis, an inflammatory disease of the eye. The prime reason for undertaking this study is based on the earlier findings that gut dysbiosis is associated with several inflammatory diseases like enterocolitis, obesity, inflammatory bowel disease and vaginosis [11–15].

Keratitis is an inflammatory disease of the eye, in which the cornea is inflamed and is often marked by moderate to intense pain, impaired eyesight and itchiness, and causes significant loss of vision [25, 26]. Keratitis is estimated to affect over a million cases annually and occurs mostly in tropical countries. In India, unilateral corneal blindness is estimated to increase to 10.6 million by 2020 [27] thus causing enormous economic burden. Keratitis normally occurs after an injury to the cornea and the causative agents of Keratitis include bacteria, fungi, viruses, and parasites [28–30]. Common organisms involved in fungal Keratitis (FK), include *Aspergillus* spp., *Fusarium* spp., *Candida* spp., *Curvularia* spp., *Penicillium* spp., *Rhizopus* spp., *Mucor* spp., and other fungi [29, 31–33]. The observed inflammation associated with Keratitis is thus attributed to the above infectious agents. But considering that gut microbiome dysbiosis could cause inflammatory diseases and other diseases remote to the gut, it would be worthwhile to investigate the possibility of dysbiosis in the gut microbiome being associated with Keratitis. Such studies would help to identify deviations from the normal core microbiome, which then could become the basis for restoration of dysbiosis by the use of probiotics, prebiotics (compounds like inulin) and specific antibiotics for manipulating the microbiota and eventually help in disease management [4, 34]. The key questions that could be addressed by studying the gut microbiomes of Keratitis individuals are: (a) are the gut microbiomes (fungi and bacteria) of normal and Keratitis individuals similar or different? and (b) is there any association between the gut microbiome and the diseased state? In this study, using fecal samples from healthy and FK individuals, we demonstrated that dysbiosis in the gut bacterial microbiome of FK patients is associated with Keratitis. However, the fungal microbiome (mycobiome) mostly remained unaltered and did not show any significant association with the disease.

## Materials and methods

### Recruitment of subjects

Thirty one healthy controls (HC) with no significant ocular pathology (15 males and 16 females) aged between 21–81 (mean 42.2 years), 32 subjects with FK (19 males and 13 females) aged between 25–69 (mean 47.1 years), attending an eye hospital, the L. V. Prasad Eye Institute, Hyderabad, India were included in the study. The participants were from two adjacent states in the southern part of India, namely Telangana (66.7%) and Andhra Pradesh (28.6%) and the remaining were from other parts of India (S1 Table). The subjects under Keratitis group included patients with microbiologically-proven fungal Keratitis based on microscopic examination of corneal scrapings. Patients diagnosed with bacterial, viral or mixed infections were excluded from the study. All participants had not taken pro- or prebiotics 3 months prior to day 1 of sample collection. The FK patients (21 out of 32) had taken oral antifungals and only one patient (FK004) had taken both antifungal and antibacterial drugs orally as part of their treatment (S1 Table). The control group (31 individuals) had not taken any antibiotics. Written informed consent was taken from all the study participants prior to sample collection and the study was approved by the institutional review board of L. V. Prasad Eye Institute, Hyderabad (Ethics Ref. No. LEC 06-14-060).

### Fecal sample collection and DNA extraction

Fecal samples were collected by the subjects in a sterile container (HiMedia, India) and stored in -80°C freezer until DNA extraction. Genomic DNA was extracted from stool samples using QIAamp DNA stool minikit (Qiagen, Hilden, North Rhine-Westphalia, Germany) following manufacturer’s instructions with a few modifications. The fecal sample in the sterile container was mixed manually using a sterile spatula (HiMedia, India) until it formed a homogeneous mixture. Then 300 mg of sample was transferred into a 2 ml centrifuge tube and extraction was carried out according to the protocol. The extraction was performed in triplicates for each sample. At the final step, DNA was eluted with 100 µl of AE buffer provided by Qiagen. Then, equal volume of DNA was taken from each replicate and pooled together for PCR amplification and sequencing. Quality of genomic DNA was checked on 0.8% agarose gel and quantification was performed using Nanodrop 8000 (GE Healthcare, India) and Qubit 2.0 fluorometer with the help of Qubit dsDNA HS Assay kit (Life Technologies, India).

### PCR amplification, Illumina library preparation and amplicon sequencing

ITS2, a region of the fungal ribosomal small subunit RNA was amplified with primers ITS3 (5'- GCATCGATGAAGAACGCAGC-3') and ITS4 (5'- TCCTCCGCTTATTGATATGC-3') [35]; whereas V3-V4 region of 16S rRNA gene of bacteria was amplified using the primers 5’-CCTACGGGNGGCWGCAG-3’ and 5’-GACTACHVGGGTATCTAATCC-3’ [36]. The amplicon libraries were prepared as per the standard Illumina protocol described by Dehingia et al., (2015) [37]. The libraries were sequenced using Illumina MiSeq 2 × 250 bp chemistry with paired-end protocol at the Xcelris Genomics Pvt. Ltd., Ahmedabad, India.

### Taxonomic classification of sequenced reads

#### Fungal microbiome

Paired-end reads corresponding to individual samples were first demultiplexed into separate files in Fastq format. Subsequently, respective paired end reads were merged into single reads based on overlapping portions, using the software FLASH, run with default options [38]. Sequences with an average Phred score below 25 were removed using the software Prinseq-lite [39]. Usearch61 was employed to remove chimeric sequences from fungal microbiome [40]. OTU picking with the retained high quality reads was performed using an ‘open reference operational taxonomic unit (OTU) picking’ approach as implemented in the QIIME pipeline [41]. UNITE OTUs (ITS) 12.11 (alpha release) clustered at 99% sequence similarity was used as reference database. UCLUST was chosen for the OTU picking (‘uclust_ref’ run with default parameters for clustering sequences with 97% similarity). Reads / OTUs (reference-OTUs) which matched the reference OTU database were assigned taxonomic lineages as provided in the database, whereas, taxonomic assignments for *denovo* clustered OTUs (denovo-OTUs) were obtained using the MOTHUR pipeline (version v.1.29.2). For this purpose, representative sequences from each of the denovo-OTUs were provided as input to the Wang Classifier [42] (bootstrap threshold of 80%). Sparse OTUs containing < 0.001% of the total number of high quality reads sequenced were removed.

#### Bacterial microbiome

The protocol for taxonomic classification of sequenced reads for bacteria with respect to filtering high quality reads, OTU picking, and taxonomy assignments was as described for fungal microbiome analysis. For chimera removal, representative sequences from the identified reference-OTUs as well as denovo-OTUs were aligned using PyNAST [43] and processed using the Chimera Slayer [44] approach implemented in QIIME (using default options). Also, GreenGenes 13.8 OTUs clustered at 97% identity was used as the reference OTU database for OTU picking and taxonomy assignment. Sparse OTUs containing < 0.001% of the total number of high quality reads sequenced were removed for constructing the final OTU abundance table. Those samples, for which approximately 40% or more of the high quality sequences could not be assigned to any OTUs, were not considered for further analysis.

All subsequent analyses (unless otherwise mentioned) were also performed separately for the bacterial and the fungal abundance tables. The OTU level abundances were also appropriately cumulated at higher levels of taxonomic hierarchy (e.g. phylum, family, genus etc.) as required for different downstream analyses.

### Diversity analyses of microbiome samples

Rarefaction curves and alpha diversity indices for the sampled microbiomes were obtained using the R-Vegan 2.4-2package (http://vegan.r-forge.r-project.org/). Three diversity indices, viz., Shannon diversity, Simpson index and number of observed OTUs were computed for each of the samples pertaining to the fungal as well as bacterial microbiomes. Subsequently, a t-test was performed to evaluate whether the alpha diversity of the microbiomes from HC were significantly different from those obtained from the subjects having FK.

### Identification of core taxonomic groups

OTUs having more than 0.01% abundance in a sample and ubiquitously present in over 80% of samples in a sample set were identified as the core OTUs for the considered sample set. Core fungal as well as bacterial OTUs were identified in the studied cohort. Core OTUs specific to the HC and FK sample sets were also identified.

### Inferring functional profile of bacterial microbiomes

Functional profiles of the bacterial microbiomes were inferred from the respective 16S taxonomic profiles, using the software PiCrust [45]. The results of PiCrust were curated in separate tables depicting the relative abundance of KEGG pathways as well as KEGG functional modules. The eukaryotic pathways / modules were removed before downstream analyses, following the implementation of the ‘removal of eukaryotic functions’ module of the software Vikodak [46].

### Identification of differentially abundant taxonomic and functional groups

Wilcoxon signed rank test was performed to identify the taxonomic groups (at all different levels of taxonomic hierarchy), in both bacterial and fungal components, which were differentially abundant in HC and FK samples (Benjamini Hochberg (BH) corrected P < 0.1 for fungal microbiome and P < 0.05 for bacterial microbiome). In addition, the bacterial functional pathways / modules discriminating between HC and FK samples were also investigated using a similar method. Given that a fraction of the subjects were undergoing treatment (antibacterial/ antifungal), the FK samples were further categorized into treated (FK_T) and untreated (FK_UT) groups. A Kruskal Wallis test was performed to assess if any of the taxonomic groups exhibited significant (BH corrected P < 0.05) differential abundance amongst HC, FK_T, and FK_UT groups of samples. Further, in order to assess whether any identified dysbiosis could be associated with the disease or be attributed to the effect of antibiotic treatment, post hoc Wilcoxon tests between the pairs of samples HC vs. FK_T, HC vs. FK_UT and FK_T vs. FK_UT were performed. PCoA plots of microbiome samples were generated (using R v3.2.5, ade4 package) based on discriminating OTUs (between HC and FK samples) identified through Wilcoxon test (BH corrected P < 0.05), using JSD as a distance metric (http://enterotyping.embl.de/enterotypes.html). A K-means clustering (k = 2) was also performed with the data used to generate the PCoA plot, and the samples adhering to the two identified clusters were indicated on the PCoA plot. Similar PCoA plot was also generated based on the differentially abundant (between HC and FK samples) KEGG functional modules corresponding to the bacterial microbiome.

### Random forest classifier for detection of FK based on microbiome signature

A random forest (RF) classifier was constructed for detecting FK based on gut microbiome composition. Abundances of all the bacterial genera identified in the HC and FK microbiome samples were used as features while building this classifier. The sample set (28 HC samples + 30 FK samples) was randomly split into training and testing set samples in the proportion 70:30, wherein the proportion of HC and FK samples were equivalent in both the training and test sets. The training procedure involved 10-fold cross-validation with 10 replicates (i.e. 100 tests) (using R v3.2.5, Random forest package v4.6.x) (https://cran.r-project.org/web/packages/randomForest/randomForest.pdf). The performance of the individual models were assessed with ‘area under curve’ (AUC) of the “receiver operating characteristics” (ROC) [47] using the R pROC package (https://cran.r-project.org/web/packages/pROC/pROC.pdf). Top 10 discriminating features were selected from each cross-validation fold and ranked based on their cumulative importance (using ‘giniscore’). To create a final ‘bagged’ model, the ranked features were progressively added (upto a maximum of 100) into the model, according to their cumulative importance, while evaluating the performance of the bagged model on the training set data (in terms of AUC) after addition of every new feature. A final ‘bagged’ RF model was arrived at which used 26 most discriminating features (genera). Efficiencies of the model on training and testing sets were plotted using the pROC package.

### Correlation network between fungal and bacterial genera

Interaction networks were generated based on pair-wise correlations between abundances of different microbial genera (both fungal and bacterial). Two separate interaction networks, one for HC samples, and another for FK samples, were built. Positive and negative interactions between all pairs of genera in a particular class of samples (HC or FK) were obtained using Spearman correlation coefficient (r). Interactions were inferred only when the effective correlation value ranged between -1 < r ≤ -0.5 (for negative interaction) or 0.5 ≤ r < 1 (for positive interaction). The microbial community correlation networks were visualized and analyzed using the softwares Cytoscape [48] and CompNet [49].

## Results

### Fungal communities inhabiting guts of FK patients and healthy subjects from India

A total of 1845 fungal OTUs (S2 Table) could be identified from 46.63 million high-quality sequenced reads (S3 Table) corresponding to fecal microbiomes from 30 HC and 32 FK subjects. Rarefaction curves showed a tendency to saturate, indicating reasonable sequencing depth and coverage for the sequenced samples (S1 Fig). It was interesting to note that the number of identified *de novo* OTUs (1470 denovo-OTUs) were significantly higher than the number of OTUs (375 reference-OTUs) for which hits could be found in the UNITE database. This is probably due to the limited number of available sequenced fungal organisms (inhabiting the human gut).

**Fig 1:**
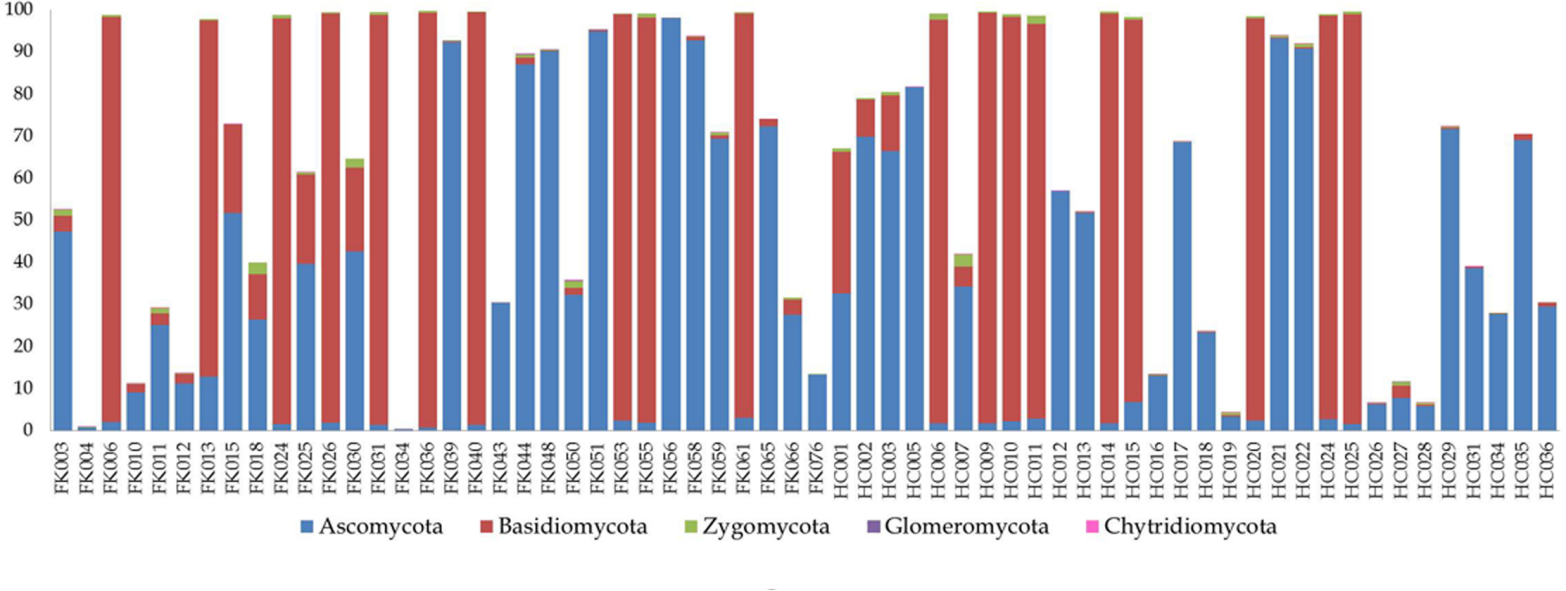
Bar plot depicting abundance of different fungal phyla in HC and FK samples.

**Fig 2:**
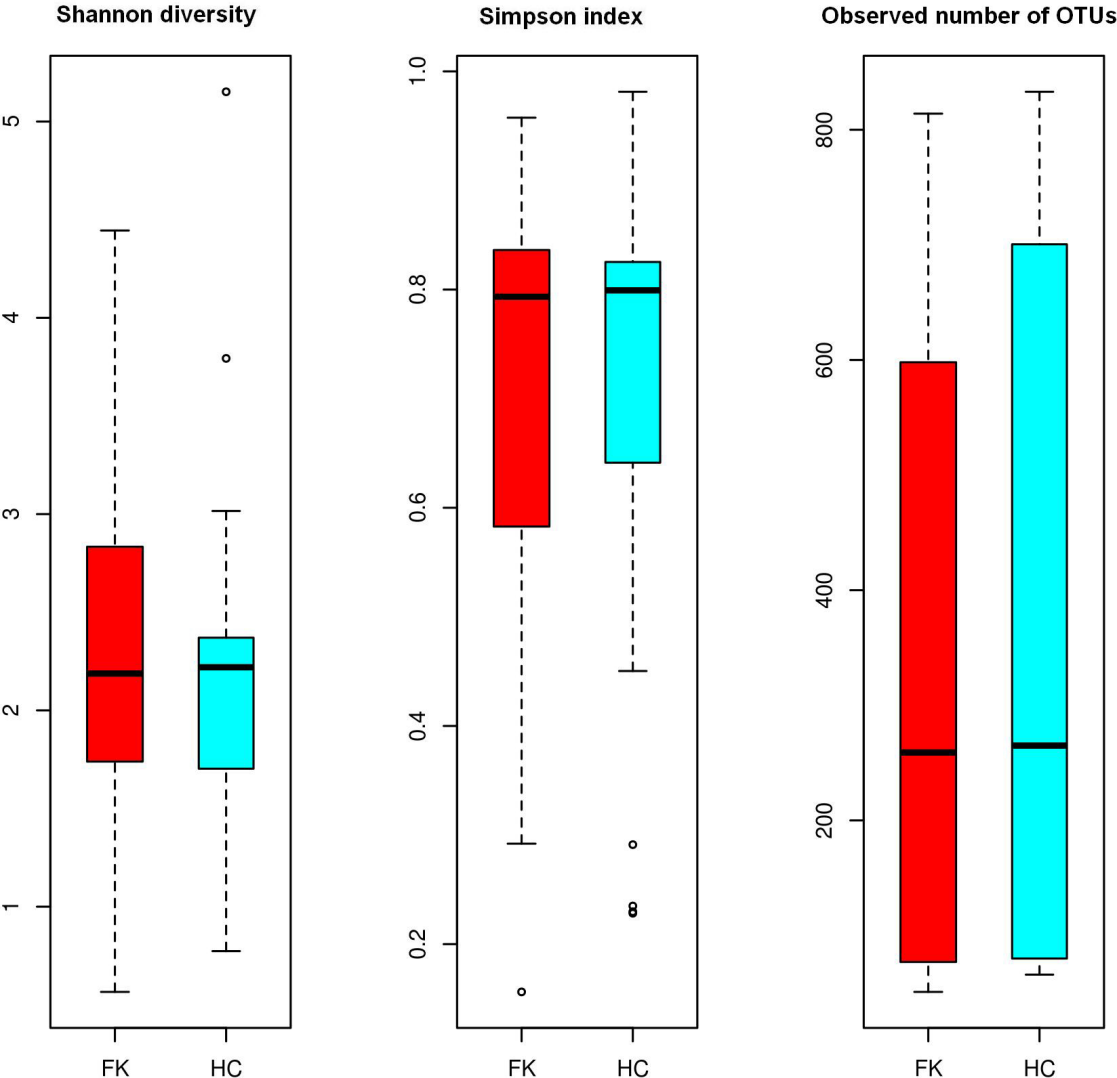
Box-plots illustrating alpha diversity indices (Shannon diversity, Simpson index and Observed OTUs) in fungal microbiomes of FK and HC samples. Median values and interquartile ranges have been indicated in the plots.

Ascomycota (mean abundance 33%) and Basidiomycota (mean abundance 32%) were observed to be the dominant phyla in both HC and FK subjects (Fig 1 and S4 Table). However, it may be noted that a significant proportion of reads (mean abundance = 34.5%) remained unclassified at the phylum level. This is in contrast to the assignment statistics at the OTU level, wherein, on average 98% of all high quality reads obtained from a sample could be clustered into either a reference-OTU or a denovo-OTU (S2 and S3 Tables). These observations indicate the presence of several hitherto unknown fungal taxonomic groups in the gut of Indian subjects and reiterate the limitations of the existing fungal sequence databases. Fig 2 depicts the alpha diversity of the mycobiomes pertaining to HC and FK samples. Both HC and FK samples exhibited equivalent number of observed OTUs and were characterized by similar Shannon and Simpson diversity measures. p-value was not statistically significant as determined by t-test.

An effort to identify core fungal OTUs (i.e. having ≥ 0.001% abundance in a sample and ubiquitously present in over 80% of the FK and HC samples) revealed only 2 OTUs, belonging to the species *Candida tropicalis* and *Candida albicans*, to be present ubiquitously across all FK samples (S5 Table). On the other hand, no core OTUs could be identified amongst the HC group of samples. Core fungal OTUs also could not be detected when all samples (HC + FK) were pooled together. Although the above mentioned *Candida* sp. have been isolated from the ocular surface of FK patients [50], the present finding of the ubiquitous presence of the two species of *Candida* in the gut of FK patients is interesting.

To examine whether the gut mycobiomes of the FK patients reflected any dysbiosis, a Wilcoxon signed rank test was performed (at both genera and OTU levels) to compare the abundances of different taxonomic groups. Only seven OTUs were observed to have statistically relevant differences (P < 0.1) in relative abundance between the FK and HC samples (Table 1). OTUs pertaining to *Candida albicans* (2 OTUs), *Aspergillus* (1 OTU), along with 3 other denovo-OTUs belonging to unclassified genera, were observed to have an enriched abundance in the FK samples. A different unclassified denovo-OTU was seen to be enriched in the HC samples. However, the overall abundance of all these apparently ‘discriminatory’ OTUs were very low (< 0.001%) and were not indicative of any significant dysbiosis. Moreover, no fungal genera were found to be significantly differentially enriched in either the HC or the FK samples (S6 Table).

**Table 1:** Discriminating fungal OTUs between HC and FK samples (BH corrected P < 0.1).

OTU ID	Taxonomic affiliation	Median Abundance (%)		Wilcoxon test P-value (BH corrected)
		HC Samples	FK Samples	
**OTUs enriched in Fungal keratitis samples**				
AB369915	*Candida albicans*	0	1.11E-03	0.095
JN851052	*Aspergillus*	0	7.78E-04	0.064
Denovo_OTU335	Unclassified	0	2.33E-04	0.064
Denovo_OTU14	Unclassified	0	1.05E-04	0.092
FJ662389	*Candida albicans*	0	7.43E-05	0.092
Denovo_OTU1686	Unclassified	0	5.84E-05	0.071
**OTUs enriched in Healthy control samples**				
Denovo_OTU1057	Unclassified	1.86E-04	0	0.064

### Gut bacterial communities in Indian FK patients and healthy subjects

Given that no significant dysbiosis could be observed in the fungal community inhabiting the gut of Indian patients suffering from FK, the bacterial components of the corresponding gut microbiomes were investigated. Out of 63 bacterial microbiomes (31 HC and 32 FK), only 58 microbiomes (28 HC and 30 FK) in which approximately 60% of the reads (ranged from 57.86% to 89.81%) assigned to an OTU were considered for further analysis (S7 Table). From 40.72 million high-quality sequenced reads (S8 Table), a total of 2115 bacterial OTUs, consisting of 1204 reference-OTUs and 911 denovo-OTUs, were identified in 58 gut microbiome samples (S7 Table), after removal of sparse and chimeric OTUs (See methods). Rarefaction plot for all the samples ensured that sequencing coverage was sufficient for making comparative analyses (S2 Fig).

**Fig 3:**
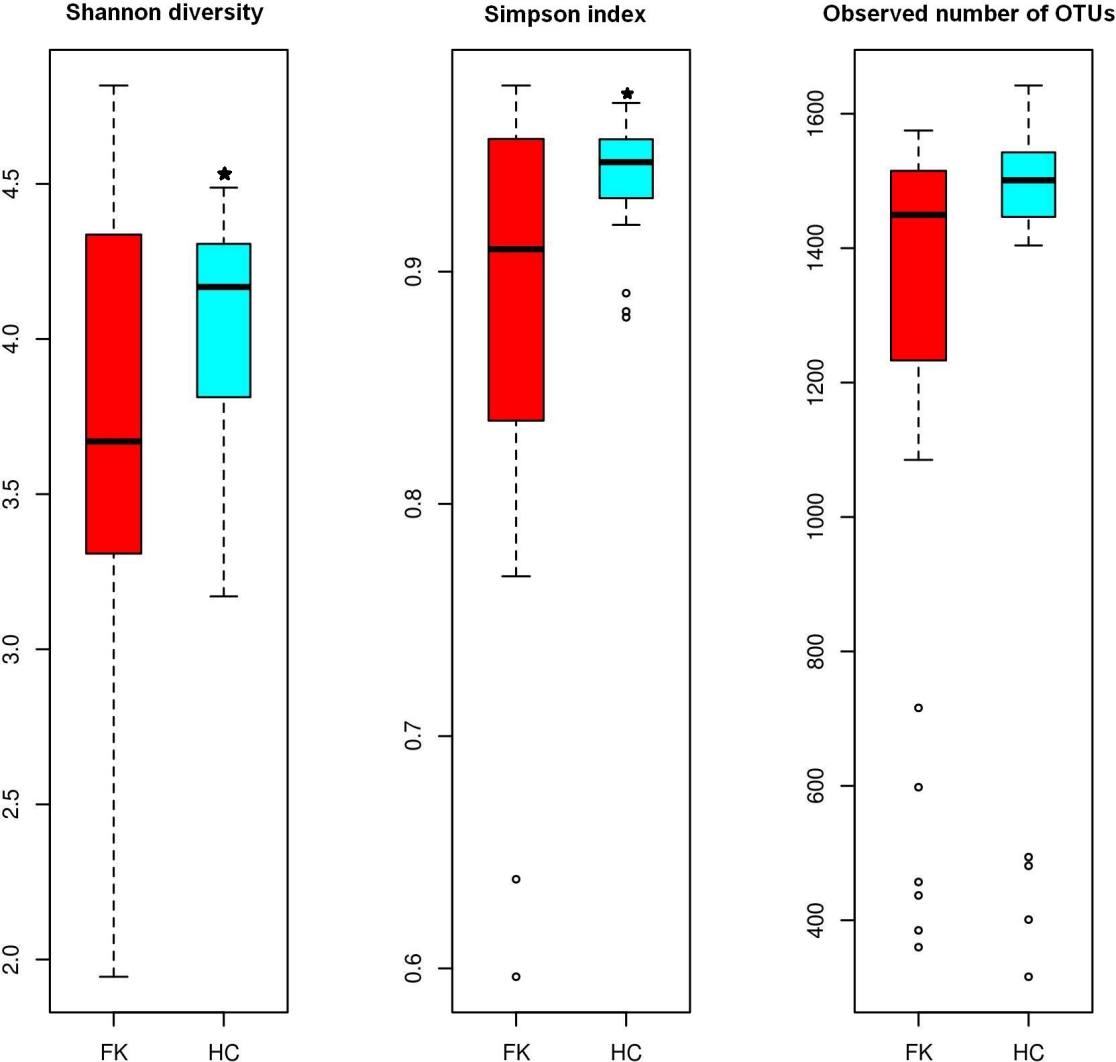
Box-plots illustrating alpha diversity indices (Shannon diversity, Simpson index and Observed OTUs) in bacterial microbiomes of FK and HC samples. Median values and interquartile ranges have been indicated in the plots. * indicates significant difference between HC and FK (p-value <0.05).

The alpha diversity of the gut bacterial communities associated with the HC and FK samples were measured in terms of number of observed OTUs per sample (richness), Simpson index (evenness) and Shannon diversity (Fig 3). While alpha diversity was observed to be higher in the HC group in terms of all compared metrics, the difference was significant for Shannon diversity (p = 0.022) and Simpson Index (p = 0.004).

**Fig 4:**
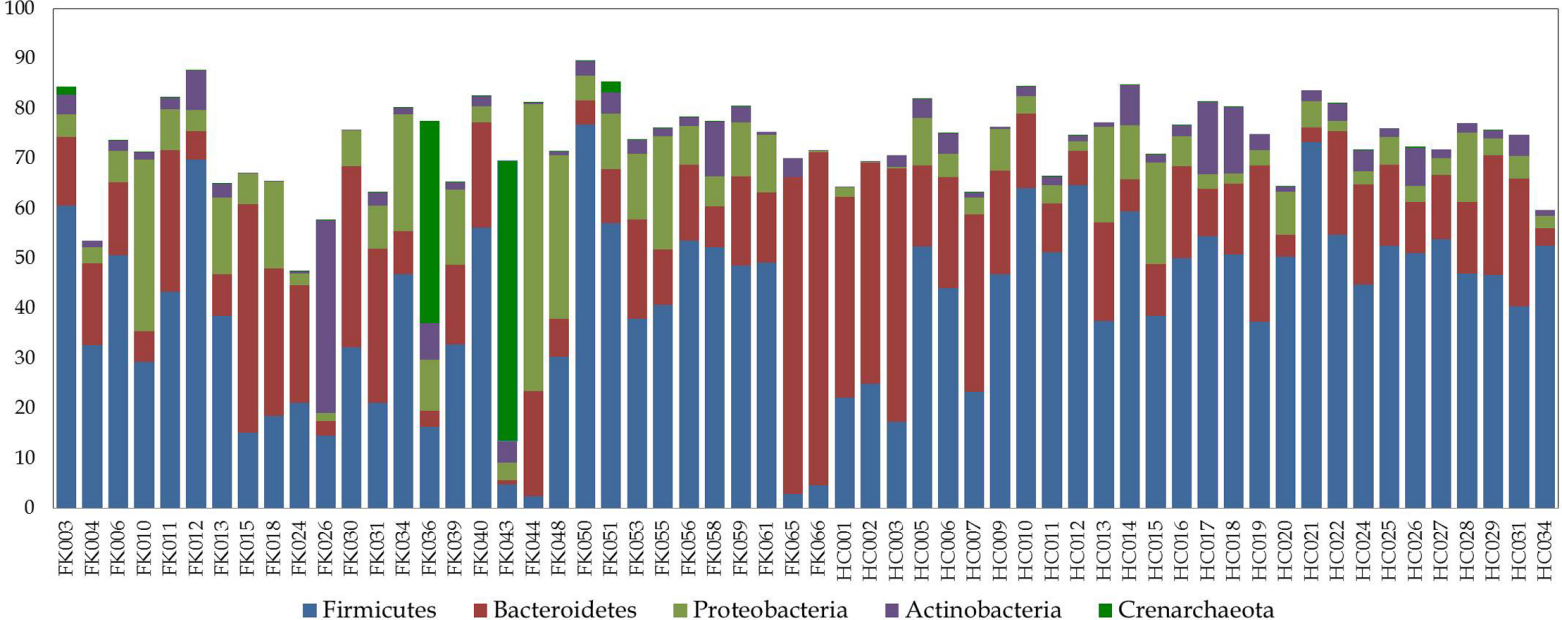
Taxonomic abundance of different bacterial phyla, across HC and FK samples. Only those phyla with > 1% mean abundance are depicted in the plot.

The gut microbiome of the sampled cohort (both FK and HC samples) was observed to be dominated by Firmicutes (Fig 4 and S9 Table), which is in accordance with earlier observations in Indian subjects [37, 51]. Three other phyla, viz. Bacteroidetes, Proteobacteria, and Actinobacteria were also observed to be present in significant abundance. A closer look revealed that while the phyla Bacteroidetes and Actinobacteria had similar abundances across FK and HC samples, the mean abundance of Firmicutes (35.35% in FK vs. 46.65% in HC, P = 0.022) and Proteobacteria (11.75% in FK vs. 5.59% in HC, P = 0.012) differed significantly (P < 0.05) across these groups. At the family level, Ruminococcaceae, Veillonellaceae, Lachnospiraceae, Prevotellaceae, and Bacteroidaceae were observed to be the most dominant taxa in the sampled cohorts (S3 Fig and S10 Table), which also confirms with earlier observations [37, 51]. However, apart from a subtle enrichment of members of the family Veillonellaceae in the HC samples, no major differences in family level abundances were apparent.

A search for core bacterial taxonomic groups could identify 66 core OTUs (ubiquitously present in over 80% of the samples with at least 0.01% abundance) when both FK and HC samples were considered (Tables 2 and S11). In addition, 57 OTUs were identified to constitute the core when only HC samples were considered (Tables 2 and S12), whereas only 12 OTUs were found to be the core for the FK group of samples (Tables 2 and S13). OTUs affiliated to members of Lachnospiraceae (11 OTUs) and Ruminococcaceae (8 OTUs) families were abundant in both HC and FK groups (S11 Table). OTUs affiliated to genus *Desulfovibri*o and species *Bacteroides fragilis* and *Ruminococcus gnavus* were identified as the core only in FK samples (S13 Table) and are not ubiquitously abundant in the HC samples. On the other hand, OTUs affiliated to the families Barnesiellaceae and Clostridiaceae, and the genera *Parabacteroides*, *Clostridium*, *Dorea*, and *Klebsiella* constituted the core of only HC samples (S12 Table). Furthermore, except OTUs of *Prevotella copri* and *Faecalibacterium prausnitzii*, all the core OTUs of HC group identified at species level were found to be exclusive to HC samples (S12 Table). All the above observations indicate subtle differences in the gut microbial communities in the FK patients as compared to the healthy individuals.

**Table 2:** Core bacterial OTUs* identified in HC and FK samples.

Taxonomic Affiliation (Family Level) of Core OTUs	Number of OTUs
**(A) Core OTUs in both HC and FK samples**	
k__Bacteria; p__Firmicutes; c__Clostridia; o__Clostridiales; f__Lachnospiraceae	18
k__Bacteria; p__Firmicutes; c__Clostridia; o__Clostridiales; f__Ruminococcaceae	15
k__Bacteria; p__Bacteroidetes; c__Bacteroidia; o__Bacteroidales; f__Bacteroidaceae	6
k__Bacteria; p__Firmicutes; c__Clostridia; o__Clostridiales; f__Veillonellaceae	4
k__Bacteria; p__Actinobacteria; c__Actinobacteria; o__Bifidobacteriales; f__Bifidobacteriaceae	3
k__Bacteria; p__Actinobacteria; c__Coriobacteriia; o__Coriobacteriales; f__Coriobacteriaceae	3
k__Bacteria; p__Bacteroidetes; c__Bacteroidia; o__Bacteroidales; f__Prevotellaceae	3
k__Bacteria; p__Firmicutes; c__Clostridia; o__Clostridiales	2
k__Bacteria; p__Firmicutes; c__Erysipelotrichi; o__Erysipelotrichales; f__Erysipelotrichaceae	2
k__Bacteria; p__Proteobacteria; c__Gammaproteobacteria; o__Enterobacteriales; f__Enterobacteriaceae	2
k__Bacteria; p__Bacteroidetes; c__Bacteroidia; o__Bacteroidales; f__[Paraprevotellaceae]	1
k__Bacteria; p__Bacteroidetes; c__Bacteroidia; o__Bacteroidales; f__Porphyromonadaceae	1
k__Bacteria; p__Bacteroidetes; c__Bacteroidia; o__Bacteroidales; f__S24-7	1
k__Bacteria; p__Firmicutes; c__Bacilli; o__Lactobacillales; f__Lactobacillaceae	1
k__Bacteria; p__Firmicutes; c__Clostridia; o__Clostridiales; f__Clostridiaceae	1
k__Bacteria; p__Proteobacteria; c__Betaproteobacteria; o__Burkholderiales; f__Alcaligenaceae	1
k__Bacteria; p__Proteobacteria; c__Deltaproteobacteria; o__Desulfovibrionales; f__Desulfovibrionaceae	1
k__Bacteria; p__Proteobacteria; c__Gammaproteobacteria; o__Aeromonadales; f__Succinivibrionaceae	1
**Total Count of Shared Core OTUs**	**66**
**(B) OTUs found to be Core only in FK samples**	
k__Bacteria; p__Firmicutes; c__Clostridia; o__Clostridiales; f__Ruminococcaceae	3
k__Bacteria; p__Firmicutes; c__Clostridia; o__Clostridiales; f__Lachnospiraceae	2
k__Bacteria; p__Bacteroidetes; c__Bacteroidia; o__Bacteroidales; f__Bacteroidaceae	2
k__Bacteria; p__Proteobacteria; c__Gammaproteobacteria; o__Enterobacteriales; f__Enterobacteriaceae	1
k__Bacteria; p__Proteobacteria; c__Deltaproteobacteria; o__Desulfovibrionales; f__Desulfovibrionaceae	1
k__Bacteria; p__Firmicutes; c__Clostridia; o__Clostridiales	1
k__Bacteria; p__Firmicutes; c__Bacilli; o__Lactobacillales; f__Streptococcaceae	1
k__Bacteria; p__Bacteroidetes; c__Bacteroidia; o__Bacteroidales; f__S24-7	1
**Total Count of Core OTUs exclusive to FK Samples**	**12**
**(C) OTUs found to be Core only in HC samples**	
k__Bacteria; p__Firmicutes; c__Clostridia; o__Clostridiales; f__Ruminococcaceae	15
k__Bacteria; p__Firmicutes; c__Clostridia; o__Clostridiales; f__Lachnospiraceae	12
k__Bacteria; p__Bacteroidetes; c__Bacteroidia; o__Bacteroidales; f__Bacteroidaceae	4
k__Bacteria; p__Firmicutes; c__Clostridia; o__Clostridiales; f__Veillonellaceae	4
k__Bacteria; p__Bacteroidetes; c__Bacteroidia; o__Bacteroidales; f__Prevotellaceae	3
k__Bacteria; p__Firmicutes; c__Clostridia; o__Clostridiales	3
k__Bacteria; p__Firmicutes; c__Clostridia; o__Clostridiales; f__Clostridiaceae	3
k__Bacteria; p__Proteobacteria; c__Gammaproteobacteria; o__Enterobacteriales; f__Enterobacteriaceae	3
k__Bacteria; p__Bacteroidetes; c__Bacteroidia; o__Bacteroidales; f__Porphyromonadaceae	2
k__Bacteria; p__Firmicutes; c__Bacilli; o__Lactobacillales; f__Streptococcaceae	2
k__Bacteria; p__Firmicutes; c__Erysipelotrichi; o__Erysipelotrichales; f__Erysipelotrichaceae	2
k__Bacteria; p__Proteobacteria; c__Betaproteobacteria; o__Burkholderiales; f__Alcaligenaceae	2
k__Bacteria; p__Bacteroidetes; c__Bacteroidia; o__Bacteroidales; f__[Barnesiellaceae]	1
k__Bacteria; p__Proteobacteria; c__Gammaproteobacteria; o__Pasteurellales; f__Pasteurellaceae	1
**Total Count of Core OTUs exclusive to HC Samples**	**57**
	

* Core OTU, an OTU present ubiquitously in a set of samples (in over 80% of the samples) with a minimum abundance (≥ 0.01%).

### Differentially abundant bacteria in the gut of FK patients compared to healthy subjects

**Fig 5:**
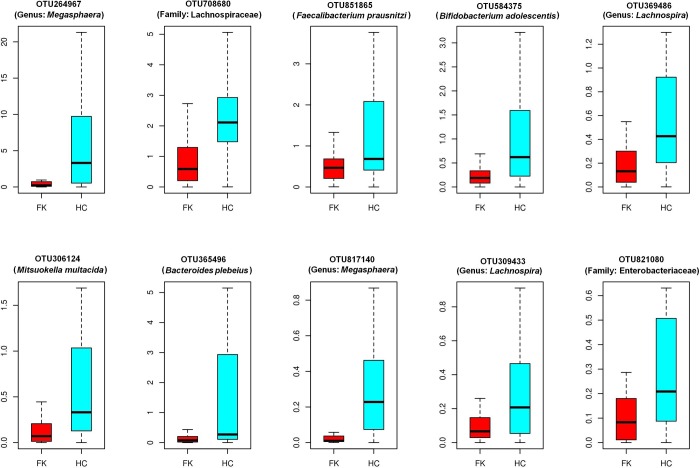
Box plots indicating relative abundance of different bacterial OTUs which exhibit significant (BH corrected P < 0.05) differential abundance across HC and FK samples. Differentially abundant OTUs having a median abundance > 0.1% in at least one group of samples has been depicted. The median abundances and the interquartile ranges have been indicated in the plots.

The overall trends in distribution of different bacterial taxonomic groups across FK and HC samples indicated possible dysbiosis of the gut microbiome in FK patients. To investigate further, Wilcoxon signed rank test was performed for identifying OTUs which exhibited significant difference in abundance (BH corrected P < 0.05) across the FK and HC samples. A total of 57 significantly differentiating OTUs could be identified (Table 3). As compared to the HC samples, the FK samples were characterized with lower abundances of 52 OTUs, whereas 5 OTUs were observed to be relatively enriched. Severe depletion of a single *Megasphaera* OTU (OTU_264967) and a single Lachnospiraceae OTU (OTU_708680) in FK samples, were most prominent amongst all other discriminating features (Fig 5). The other taxonomic groups that could discriminate FK from HC samples include *Faecalibacterium prausnitzii* (OTU_851865), *Bifidobacterium adolescentis* (OTU_584375), Lachnospira (OTU_369486 and OTU_309433), *Mitsuokella multacida* (OTU_306124), and *Bacteroides plebeius* (OTU_365496), all of which were relatively enriched in HC samples (Table 3). On the other hand, 5 OTUs affiliated to *Bacteroides fragilis* (species), *Dorea* and *Treponema* (genera), Fusobacteriaceae (Family), and Acidimicrobiales (order) were found to have significantly higher abundance in FK samples. Not all of the discriminating OTUs could be identified at the species level, making it difficult to comment on their function role. Amongst those that could be assigned at the species level was *Faecalibacterium prausnitzii*, a known anti-inflammatory bacterium, whose enriched abundance in HC patients could be imparting some beneficial effects. In contrast, *Treponema* and *Bacteroides fragilis* [52] are gut pathogens and are abundant in the FK patients, playing a potential pro-inflammatory role.

**Table 3:** Bacterial OTUs exhibiting significant (BH corrected P < 0.05) differential abundance across HC and FK samples.

**OTU ID**	**Taxonomic affiliation**	**Median Abundance (%)** *		**Wilcoxon test -P value** **(BH corrected** **P- value < 0.05)**
		**HC Samples**	**FK Samples**	
**OTUs enriched in Fungal Keratitis samples**				
OTU2200896	*Bacteroides fragilis*	0.01	0.07	0.007
OTU659361	*Dorea*	0.00	0.02	0.007
OTU514045	*Treponema*	0.00	0.01	0.003
OTU298592	[Family] Fusobacteriaceae	0.00	0.07	0.028
OTU849612	[Order] Acidimicrobiales	0.00	0.01	0.008
**OTUs enriched in Healthy control samples**				
OTU185659	*Acidaminococcus*	0.01	0.00	0.005
OTU183480	*Bacteroides*	0.22	0.04	0.008
OTU339013	*Bacteroides*	0.05	0.01	0.028
OTU364903	*Bacteroides*	0.02	0.00	0.019
OTU365496	*Bacteroides plebeius*	0.35	0.08	0.007
OTU584375	*Bifidobacterium adolescentis*	0.67	0.19	0.019
OTU1105343	*Clostridium*	0.07	0.02	0.046
OTU583746	*Dialister*	0.14	0.01	0.021
OTU523542	*Dorea*	0.03	0.01	0.015
OTU851865	*Faecalibacterium prausnitzii*	0.68	0.47	0.045
OTU813217	*Klebsiella*	0.11	0.05	0.027
OTU369486	*Lachnospira*	0.43	0.13	0.001
OTU309433	*Lachnospira*	0.23	0.07	0.015
OTU314095	*Lachnospira*	0.17	0.04	0.001
OTU349257	*Lachnospira*	0.17	0.02	0.001
OTU264967	*Megasphaera*	3.98	0.22	0.002
OTU817140	*Megasphaera*	0.24	0.01	0.001
OTU298050	*Megasphaera*	0.14	0.01	0.006
OTU266210	*Megasphaera*	0.06	0.01	0.001
OTU151623	*Megasphaera*	0.01	0.00	0.033
OTU149335	*Mitsuokella*	0.03	0.00	0.001
OTU687245	*Mitsuokella*	0.01	0.00	0.001
OTU306124	*Mitsuokella multacida*	0.41	0.07	0.005
OTU531436	*Roseburia*	0.14	0.04	0.000
OTU4332082	*Roseburia*	0.02	0.00	0.009
OTU328892	*Roseburia*	0.01	0.00	0.013
OTU362947	*Roseburia faecis*	0.02	0.01	0.015
OTU304211	*Ruminococcus*	0.12	0.01	0.013
OTU344523	*Ruminococcus*	0.07	0.01	0.016
OTU215097	*Sutterella*	0.06	0.00	0.002
**OTU ID**	**Taxonomic affiliation**	**Median Abundance (%)** *		**Wilcoxon test -P value** **(BH corrected** **P- value < 0.05)**
		**HC Samples**	**FK Samples**	
OTU315846	[Family] Barnesiellaceae	0.05	0.01	0.005
OTU780650	[Family] Clostridiaceae	0.01	0.00	0.021
OTU366392	[Family] Coriobacteriaceae	0.04	0.00	0.017
OTU816299	[Family] Coriobacteriaceae	0.01	0.00	0.001
OTU821080	[Family] Enterobacteriaceae	0.23	0.08	0.027
OTU581021	[Family] Enterobacteriaceae	0.01	0.00	0.004
OTU708680	[Family] Lachnospiraceae	2.11	0.59	0.005
OTU369027	[Family] Lachnospiraceae	0.19	0.10	0.042
OTU211935	[Family] Lachnospiraceae	0.05	0.01	0.003
OTU338992	[Family] Lachnospiraceae	0.04	0.02	0.005
OTU186968	[Family] Lachnospiraceae	0.01	0.00	0.036
OTU846141	[Family] Lachnospiraceae	0.01	0.01	0.022
OTU176306	[Family] Lachnospiraceae	0.01	0.00	0.004
OTU191332	[Family] Ruminococcaceae	0.19	0.03	0.010
OTU355685	[Family] Ruminococcaceae	0.13	0.05	0.026
OTU539328	[Family] Ruminococcaceae	0.05	0.02	0.024
OTU359175	[Family] Ruminococcaceae	0.03	0.01	0.030
OTU350121	[Family] Ruminococcaceae	0.01	0.00	0.012
OTU820764	[Family] Veillonellaceae	0.01	0.00	0.000
OTU4429981	[Order] Clostridiales	0.02	0.00	0.005
OTU357471	[Order] Clostridiales	0.02	0.01	0.013
OTUdenovo11856^**#**^	*Lactobacillus ruminis*	0.12	0.02	0.003

**#** Denovo OTU

* Differentially abundant OTUs having a median abundance > 0.01% in at least one group of samples are listed

**Fig 6:**
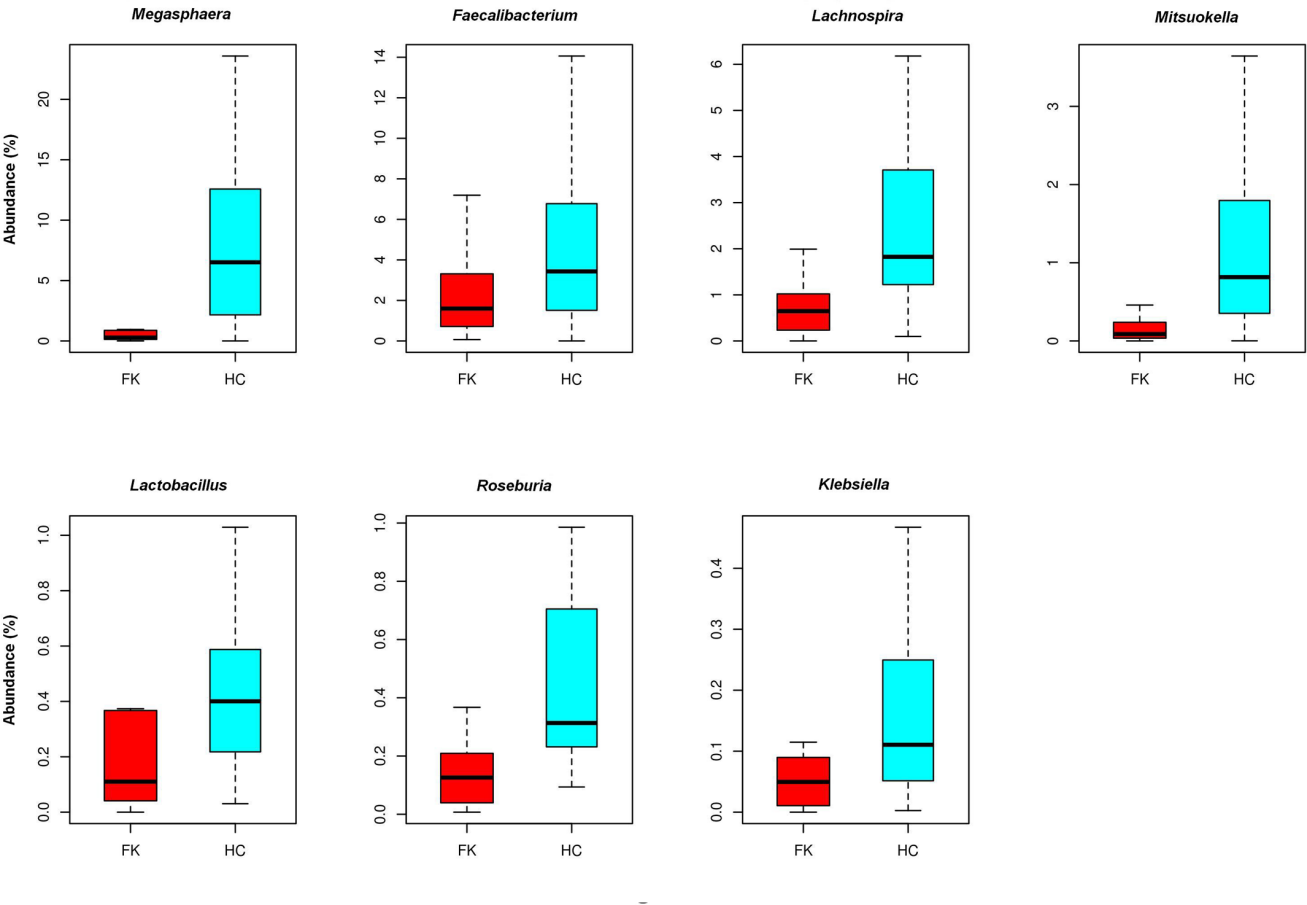
Box plots indicating relative abundance of different bacterial genera which exhibited significant (BH corrected P < 0.05) differential abundance across HC and FK samples. Differentially abundant genera having a median abundance > 0.1% in at least one group of samples has been depicted. Median abundances and interquartile ranges have been indicated in the plots.

The difference in gut bacterial microbiome composition between FK and HC samples were also apparent at the bacterial genera level. Fig 6 depicts the relative abundances of the most discriminating bacterial genera between HC and FK samples. *Megasphaera*, *Lachnospira and Faecalibacterium* were observed to be the most abundant genera in the HC samples, which showed a significant depletion in the FK samples.

**Fig 7:**
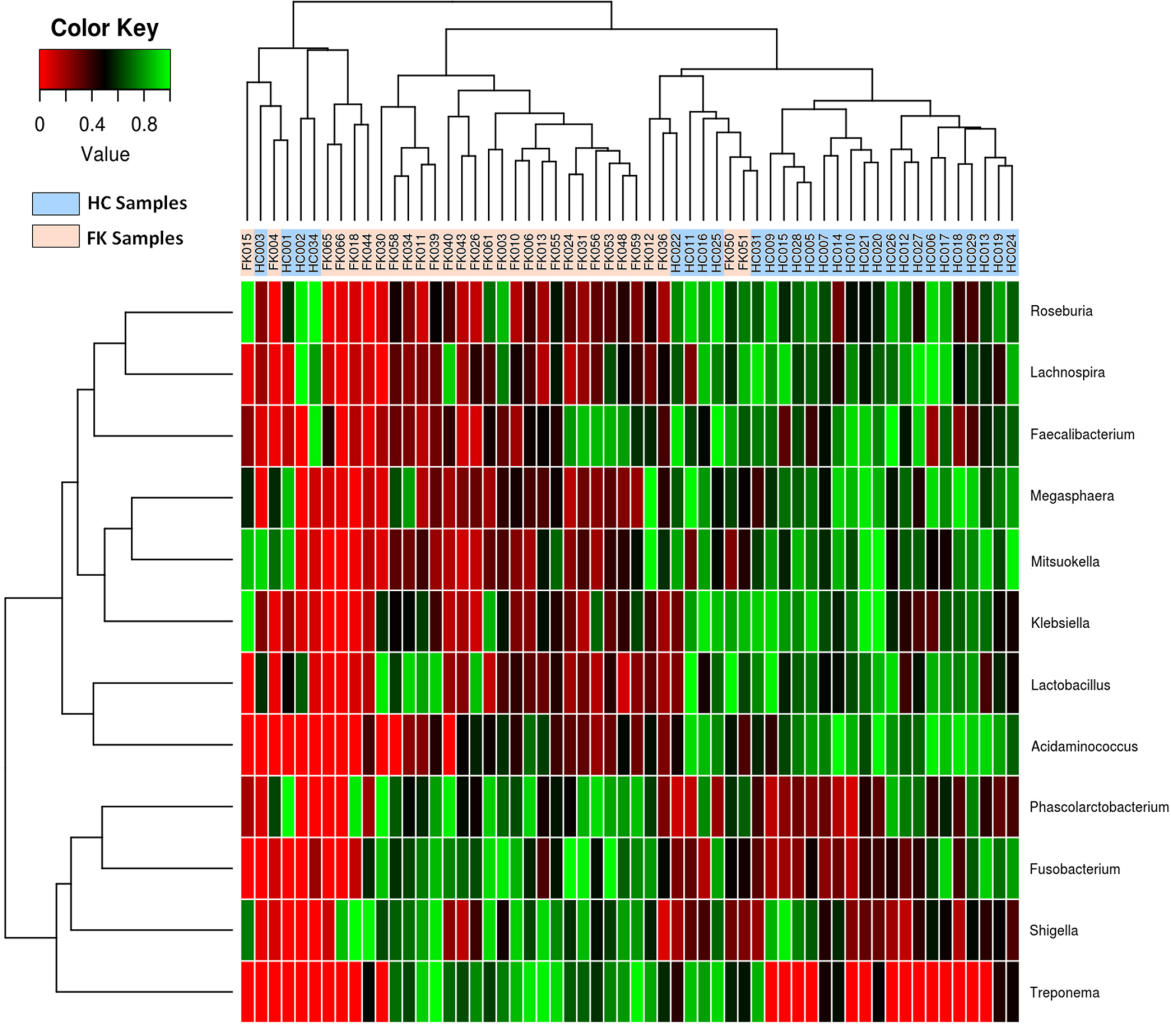
Two dimensional heatmap depicting rank normalized abundances (scaled between 0 and1) of 12 bacterial genera which were significantly enriched either in HC or FK samples. The discriminating genera, as well as the samples (HC and FK) have been arranged along the two dimensions (axes) based on hierarchical clustering.

A two-dimensional heatmap (Fig 7), depicting the rank-normalized abundances (scaled between 0 and 1) of the differentially abundant bacterial genera, further illustrates the distinct patterns of gut bacterial composition in FK and HC samples. A hierarchical clustering based on the depicted rank normalized abundances of different genera could sufficiently segregate HC and FK samples. On the other hand, two distinct groups of bacterial genera could be identified, which exhibited contrasting abundance trends in the FK and HC samples. The group consisting of genera that were found to be enriched in HC samples included *Roseburia*, *Lachnospira*, *Faecalibacterium*, *Megasphaera*, *Mitsuokella*, *Klebsiella*, *Lactobacillus* and *Acidaminococcus*. The other group, showing a contrasting trend, consisted of the genera *Phascolarctobacterium*, *Fusobacterium*, *Shigella* and *Treponema*. Interestingly, several species belonging to the genera constituting the latter group are known to cause infection [53–55].

**Fig 8:**
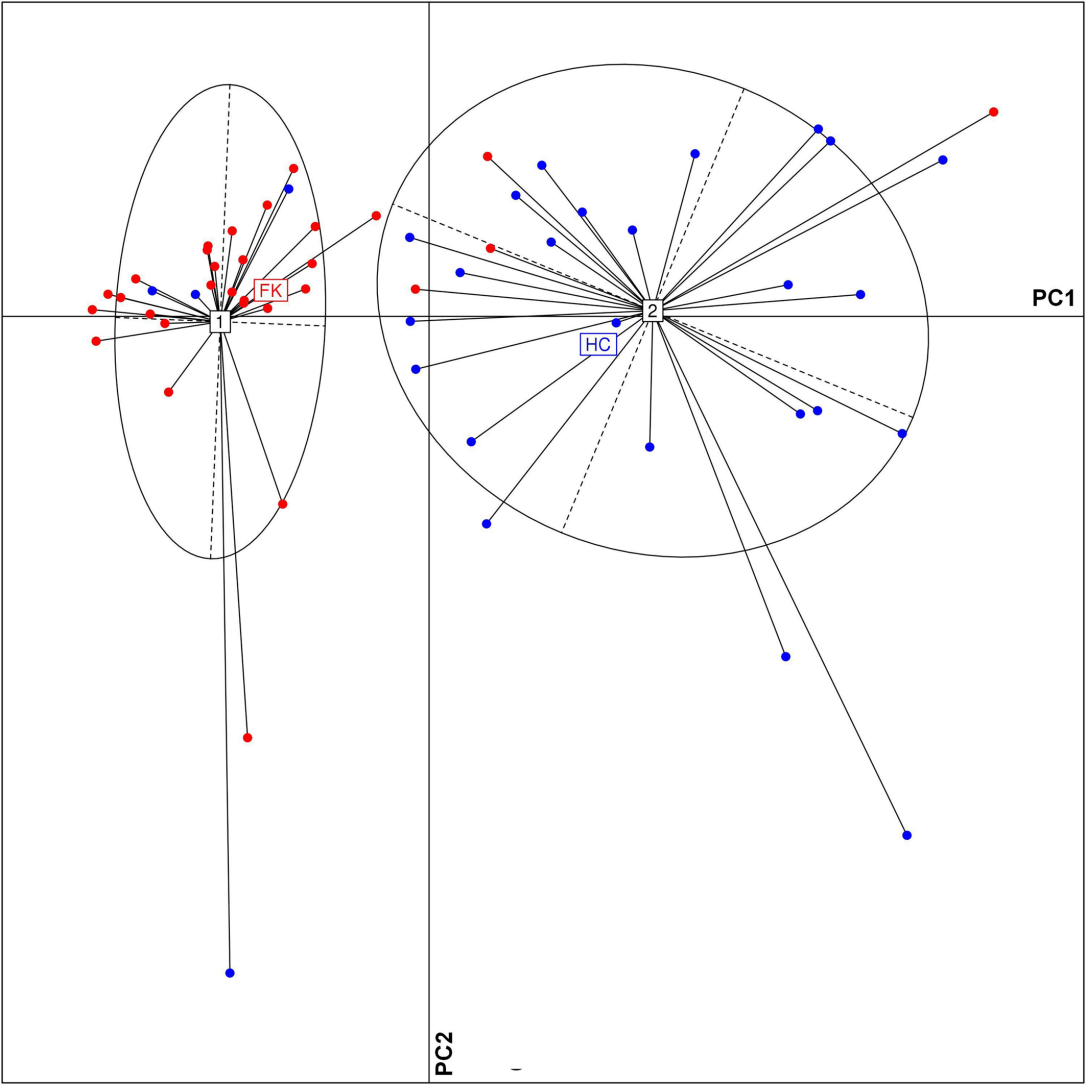
Principal Coordinate Analysis (PCoA) based on JSD distances between bacterial OTU abundance profiles of different FK (red) and HC (blue) microbiome samples. Samples plotted along first two principal coordinates showed distinct clustering of HC and FK samples.

A PCoA plot of the FK and HC samples, based on the relative abundance of discriminating OTUs, also showed distinct clusters enriched with either HC or FK samples (Fig 8). 26 out of the 30 samples belonging to FK group clustered together on the PCoA plot. Similarly, 24 out of 28 samples from the HC group formed a separate cluster. The relatively bigger spread of the HC samples in the plot can probably be linked to the higher taxonomic diversity of the HC associated bacterial microbiome.

Considering that some of the FK subjects were undergoing treatment (antibacterial/ antifungal), the FK samples were further categorized into treated (FK_T) and untreated (FK_UT) groups. A Kruskal Wallis test was performed to assess, which of the taxonomic groups exhibited significant (BH corrected P < 0.05) differential abundance amongst HC, FK_T, and FK_UT groups of samples (S14 Table). Further, to assess whether the identified dysbiosis could be associated with the disease or could be attributed to the effect of treatment, post hoc Wilcoxon tests between the pairs of samples viz., HC vs. FK_T, HC vs. FK_UT and FK_T vs. FK_UT were performed (S14 Table). The results did not indicate any significant differences between the FK_UT and FK_T samples, and reiterated that the FK disease could be associated with a dysbiotic gut bacterial community.

### Functional profiles of gut bacterial communities associated with FK patients and healthy subjects

**Fig 9:**
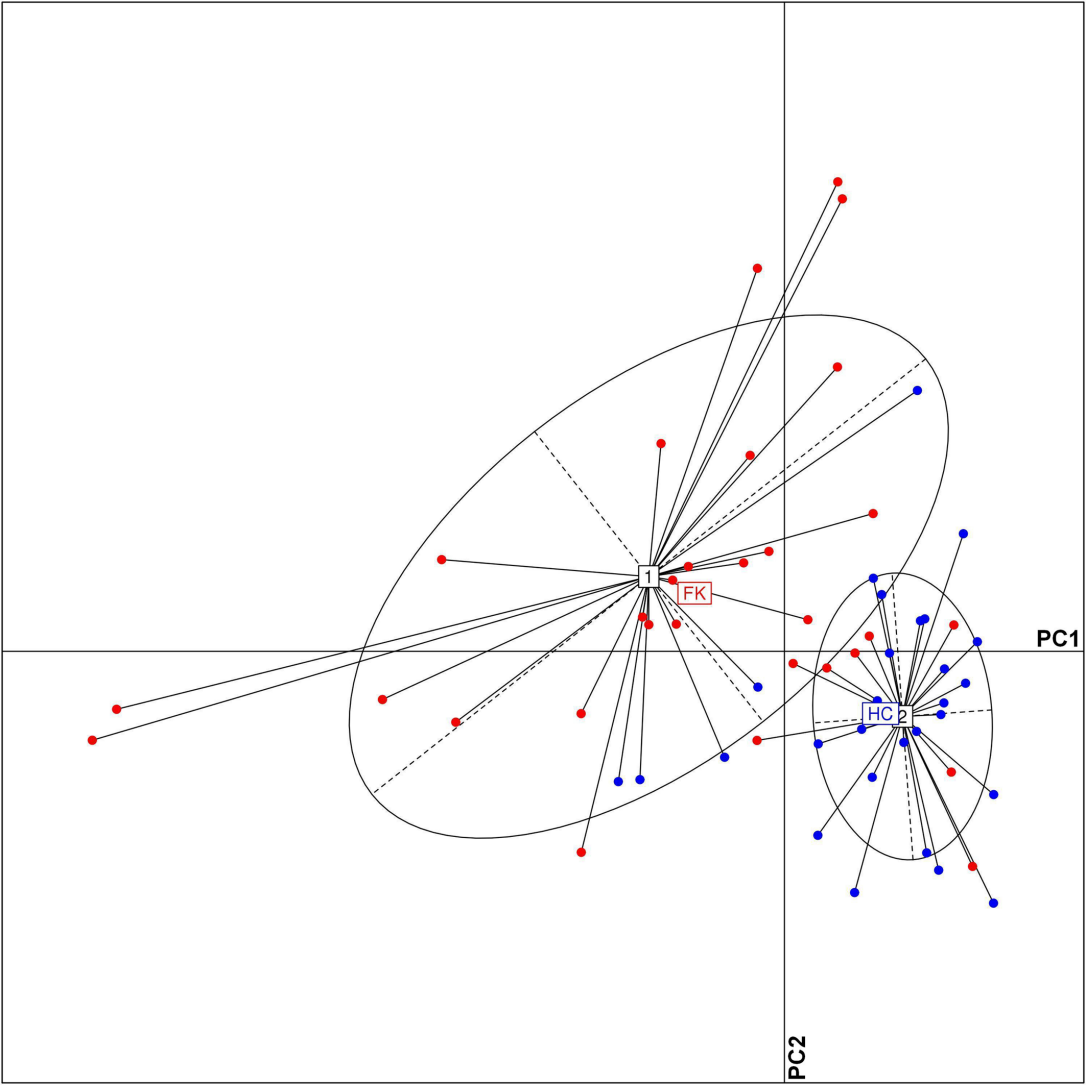
Principal Coordinate Analysis (PCoA) based on functional abundance profiles (KEGG modules) of FK (red) and HC (blue) microbiome samples. Samples plotted along first two principal coordinates showed distinct clustering of HC and FK samples.

Functional profiles of the bacterial microbiome samples in terms of KEGG pathways (S15 Table) and KEGG modules (S16 Table), were inferred from the respective 16S taxonomic profiles. Wilcoxon signed rank test was performed to identify the bacterial pathways/ modules which were differentially abundant in HC and FK samples (BH corrected P < 0.05) (Tables 4 and S17 and S18). Three KEGG pathways, namely, Endocytosis, Indole alkaloid biosythesis and Betalain biosynthesis, were observed to be significantly enriched in FK samples. The Lipid Metabolism, Carbohydrate Metabolism and Signal Transduction pathways were also seen to have relatively higher abundances in FK samples. On the other hand, six KEGG pathways, which were observed to be enriched in the HC group included Metabolism of Cofactors and Vitamins, Amino Acid Metabolism, Energy Metabolism, Cellular Processes and Signaling, Metabolism of Terpenoids and Polyketides, and Biosynthesis of Other Secondary Metabolites. A PCoA plot of the FK and HC samples, based on their functional profiles (utilizing discriminating KEGG functional modules), also showed distinct HC and FK sample associated clusters (Fig 9). However, it was worthy to note that in contrast to the PCoA plot corresponding to taxonomic abundance (Fig 8), the HC cluster in the functional abundance based PCoA plot was observed to be spread out over a much smaller area. This could be indicative of the fact that despite being more diverse in terms of taxonomy, the HC microbiome is probably more coherent in its functional aspect, as compared to the FK samples.

**Table 4:** Discriminating functional pathways (KEGG) between HC and FK samples.

KEGG Pathway (At Functional Hierarchy level 3)	Log_2_ Fold change	Wilcoxon test—P value (BH–corrected P-value < 0.05 by Wilcoxon test)	KEGG Functional Hierarchy level 1	KEGG Functional Hierarchy level 2
**Pathways enriched in FK samples**				
Endocytosis	13.041	0.002	Cellular Processes	Transport and Catabolism
Indole alkaloid biosynthesis	4.378	0.018	Metabolism	Biosynthesis of Other Secondary Metabolites
Betalain biosynthesis	4.335	0.017	Metabolism	Biosynthesis of Other Secondary Metabolites
Biosynthesis of unsaturated fatty acids	0.181	0.029	Metabolism	Lipid Metabolism
Phosphatidylinositol signaling system	0.113	0.033	Environmental Information Processing	Signal Transduction
Propanoate metabolism	0.104	0.034	Metabolism	Carbohydrate Metabolism
Fatty acid biosynthesis	0.103	0.038	Metabolism	Lipid Metabolism
**Pathways enriched in HC samples**				
Pantothenate and CoA biosynthesis	-0.047	0.015	Metabolism	Metabolism of Cofactors and Vitamins
Phenylalanine, tyrosine and tryptophan biosynthesis	-0.052	0.037	Metabolism	Amino Acid Metabolism
Carbon fixation in photosynthetic organisms	-0.072	0.015	Metabolism	Energy Metabolism
Other ion-coupled transporters	-0.112	0.038	Unclassified	Cellular Processes and Signaling
Biosynthesis of ansamycins	-0.191	0.012	Metabolism	Metabolism of Terpenoids and Polyketides
Butirosin and neomycin biosynthesis	-0.193	0.038	Metabolism	Biosynthesis of Other Secondary Metabolites

### Predictive model for FK based on microbiome signature

**Fig 10:**
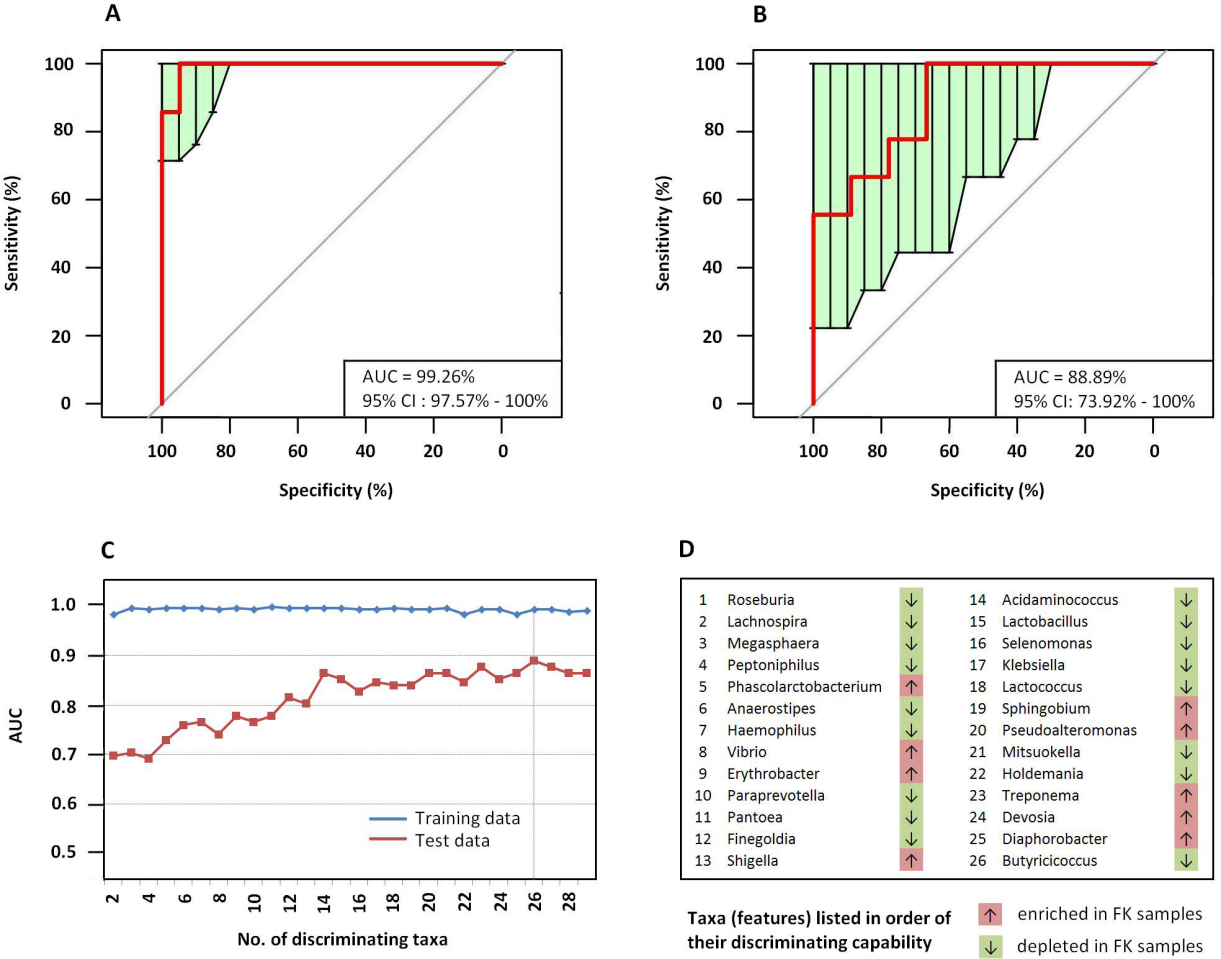
Random forest classifier for microbiome based detection of fungal Keratitis.

A RF classifier was constructed for prediction of FK based on microbiome signatures pertaining to the gut bacterial communities. The samples were randomly split into training and testing sets for the purpose. After training and repeated cross-validation (details in Methods) a final classifier (‘bagged’ RF model) was built, which could detect a FK sample based on abundance values of 26 highly discriminating genera between HC and FK samples (Fig 10). The area under the ROC curve (AUC of ROC) for the trained ‘bagged’ model, attained a near perfect value of 99.26%. Assessing the efficiency of the RF classifier with the test set samples indicated a high AUC value of 88.89%. Apart from being a potential tool for microbiome based diagnosis of FK, this model also provides evidence in favour of the observed dysbiosis in FK associated gut microbiomes.

### Interactions between fungal and bacterial groups inhabiting the gut of FK patients and healthy subjects

**Fig 11:**
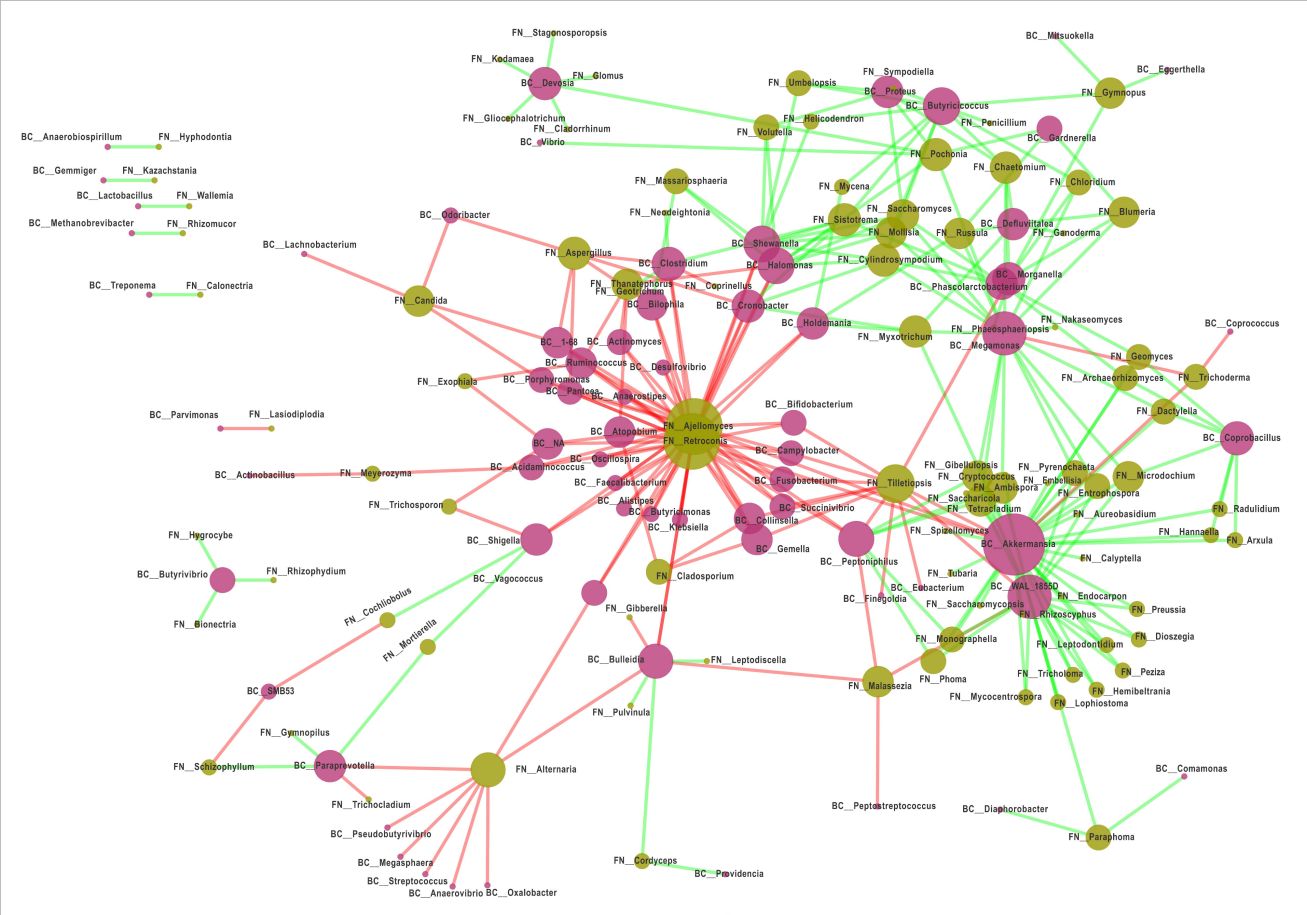
Bacteria-Fungi interaction network for the HC samples (based on correlation of genera-level abundance). The node sizes in the network correspond to their degree. The bacterial genera have been highlighted as red nodes, whereas the fungal genera have been highlighted as green nodes. The positive and negative correlations / interactions have been indicated with green edges and red edges respectively.

**Fig 12:**
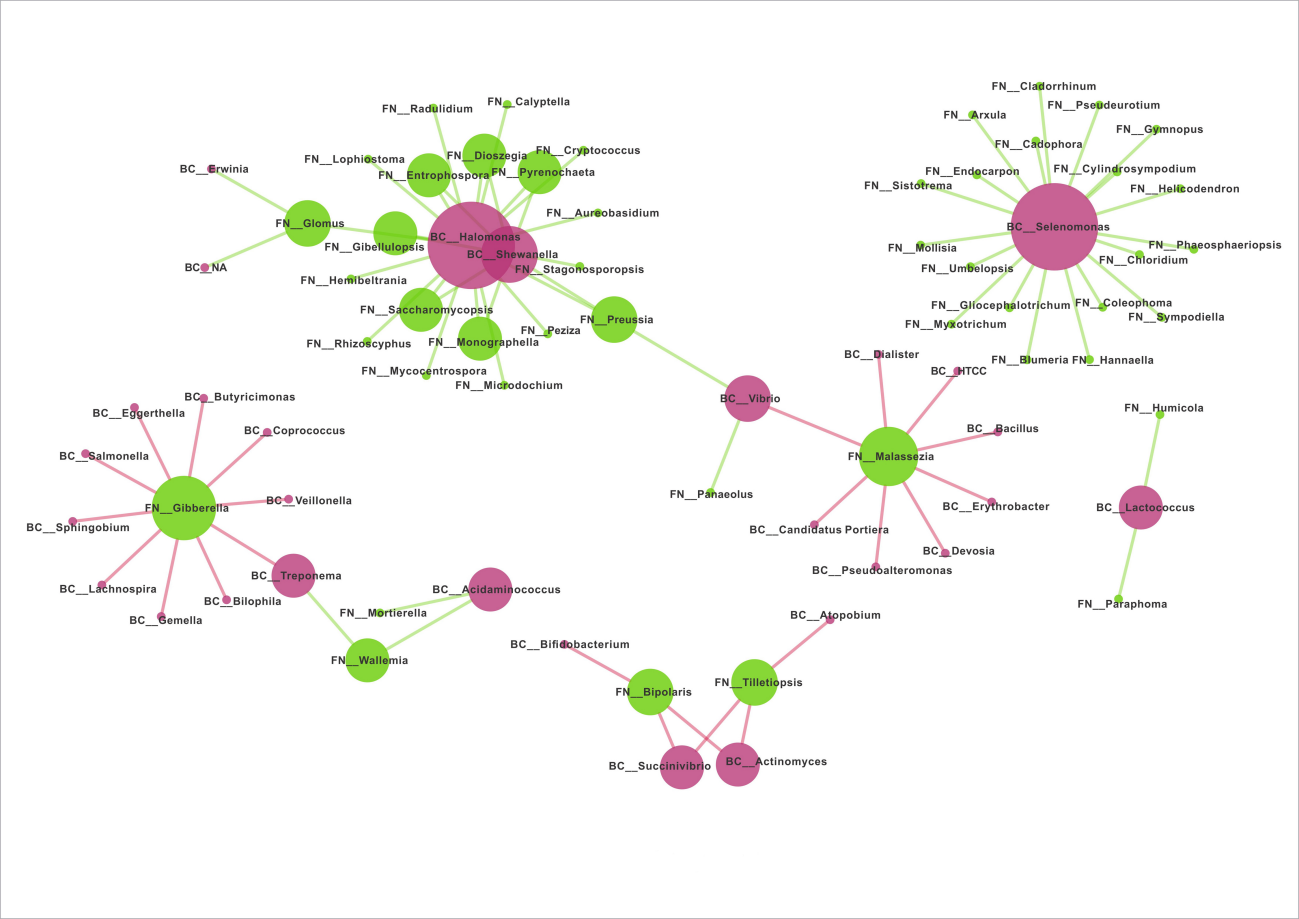
Bacteria-Fungi interaction network for the FK samples (based on correlation of genera-level abundance). The node sizes in the network correspond to their degree. The bacterial genera have been highlighted as red nodes, whereas the fungal genera have been highlighted as green nodes. The positive and negative correlations / interactions have been indicated with green edges and red edges respectively.

Interaction networks were generated based on pair-wise correlations between abundances of different microbial genera (both fungal and bacterial). Two separate interaction networks, one for HC samples, and another for FK samples were built (see Methods). A single large and well-connected network consisting of both bacterial as well as fungal genera could be observed in case of HC samples (Fig 11). In contrast, the FK network was observed to be smaller in size and consisted of multiple small disjoint networks (Fig 12). However, in both cases, certain ‘hub’ genera (with high degree) could be identified. Interestingly, the bacterial hub genera, in case of both HC and FK networks, were observed to be positively correlated with multiple fungal genera. On the other hand, the fungal hub genera were negatively correlated with the bacterial genera in both HC and FK networks.

## Discussion

Keratitis, an inflammatory disease of the eye caused by fungi, bacteria, viruses or parasites, has been reported to be the most common cause of corneal blindness, especially in the rural population and in countries with lower socioeconomic status [56], thus leading to a huge economic burden to the developing world.

There are increasing evidences linking gut microbiome to human diseases not only related to intestines but to diseases of several extra-intestinal organs including liver, lungs, heart, brain etc. [57–62]. Studies linking the gut microbiome with ocular diseases are rare. Recent studies have demonstrated association of the gut microbiome with severity of ocular mucosal disease in Sjögren syndrome (SS) patients [24], with Uveitis patients [63], Uveitis in Bechet’s disease patients [64] and in mice [18, 19] and with age-related macular degeneration in AMD patients and mice [23]. All these findings suggest a significant role for the gut microbiome in ocular diseases. But, the exact mechanism is still unclear though it is possibly mediated by the interaction of the microbiota of the microbiome through their metabolic products with the host immune system. Kugadas et al. (2016; 2017) [65, 66] demonstrated that gut commensals confer broader protection to the eye by regulating the immune response at the ocular surface by modulating secretory IgA levels and IgA transcripts and thus regulating the susceptibility to ocular keratitis. The same group also demonstrated that *Corynebacterium mastitidis* that lives on the conjunctiva alters local mucosal immunity and protects the eye from pathogenic infection [67].

Dysbiosis, the alteration in gut microbiome, has been implicated in several of these diseases as the primary indication of the diseased state. In this study, we have compared the gut microbiomes of FK subjects and HC using high-throughput Illumina sequencing of 16S and ITS amplicons from stool samples to identify dysbiosis (if any) in the gut microbiome of FK patients. The very high sequencing depth in this study is expected to sufficiently capture the diversity of the microbiomes, which was also indicated by the rarefaction analyses. Even after stringent quality filtering, an average of 752073and 674150 high quality reads per sample could be obtained for fungal and bacterial microbiomes respectively. To the best of our knowledge, no previous studies have investigated the bacterial and fungal microbiomes with such high sequencing depth in a single study.

Analysis of the gut fungal microbiomes indicated that fungi affiliated with the phyla Ascomycota and Basidiomycota were predominant and this concurs with study by Bhute et al., [51], which is the only other study on Indian gut fungal microbiome. A significant proportion of reads (mean abundance = 34.5%) remained unclassified at the phylum level in both the HC and FK individuals reiterating the limitations of the existing fungal sequence databases. Further the alpha diversity of the mycobiomes in the HC and FK samples exhibited similar Shannon and Simpson diversity measures. No core OTU was detected in HC samples but *Candida tropicalis* and *Candida albicans*, constituted the core microbiome of FK group. Both these are pathogenic yeast species reported in many diseases, especially candidiasis [68]. Further comparison of the gut fungal microbiomes of HC and FK individuals indicated that *Candida albicans* (2 OTUs), *Aspergillus* (1 OTU), along with 3 other denovo-OTUs belonging to unclassified genera, were enriched in the FK samples. However, the overall abundance of all these apparently ‘discriminatory’ OTUs were very low (< 0.001%) and were not indicative of any significant dysbiosis. Moreover, no fungal genera were found to be significantly differentially enriched in either the HC or the FK samples (S6 Table). Thus in FK individuals, the fungal microbiome did not differ from HC individuals.

This is the first study demonstrating dysbiosis in gut bacterial microbiome in FK subjects (irrespective of whether they were treated with antifungal agents) compared to HC, thus implying a clinical significance of gut microbiome in the pathophysiology of ocular diseases. Statistical tests were performed to ensure that the observed dysbiosis was not an artefact arising due to the treatment of FK subjects with antifungal agents. The reasons as to why there was no significant change in the bacterial microbiomes of FK subjects when exposed to antifungals (azole drugs) may be attributed to the observation that some of these azoles like Ketaconazole and Voriconazole reach the feces as inactive metabolites, or their elimination is by renal excretion as in the case of Fluconazole or by both renal and bile as in the case of Itraconazole [69, 70] and because they are specific to fungi.

In accordance with a recent study [51] on the gut microbial communities in Indian subjects, we also observed that gut microbiome of HC is highly enriched with bacteria affiliated to the families Veillonellaceae, Ruminococcaceae, and Lachnospiraceae including genus *Megasphaera* which constitutes >5% of the total bacterial abundance in HC. Lachnospiraceae and Ruminococcaceae are known producers of short-chain fatty acids (SCFAs) like acetate, propionate, and butyrate that are involved in promoting several health benefits [71, 72] including anti-inflammatory response.

Our data also demonstrates a reduction in abundance and diversity in the microbiomes of Keratitis patients similar to that observed in patients with other diseases such as stage 4 Hepatitis C [73] and myalgic encephalomyelitis/chronic fatigue syndrome [74] which are chronic inflammatory diseases. But there are several other diseases which also show similar reduction in diversity as in inflammatory bowel diseases [75–77], Sjögren Syndrome [24] and Alzheimer’s disease [78]. Our data re-emphasises that microbiomes of healthy controls are more abundant and diverse than the diseased state gut microbiomes. It is possible that the observed reduction in microbiome diversity or the abundance of particular taxa is responsible for the diseased state. This prediction would be acceptable only if one could demonstrate a protective role of the bacteria that have decreased in FK subjects. In fact this may indeed be the case since SCFA (particularly butyrate) producing bacteria, which are involved in promoting several health benefits [71, 72] like those affiliated to Lachnospiraceae and Ruminococcaceae and to the genera *Megasphaera*, *Roseburia*, *Lachnospira*, *Acidaminococcus*, and *Mitsuokella multacida* [79–81] were decreased in FK individuals compared to the HC individuals. In addition, pro-inflammatory Enterobacteriaceae and the pathogenic genera *Shigella* and *Treponema* were increased in FK subjects. *Shigella* is associated with reduction in butyrate-producing bacteria [79]. It was also observed that the GI tract commensal bacterium *Bacteroides fragilis* is enriched in FK subjects. *B*. *fragilis* is a potential opportunistic pathogen and its lipopolysaccharides is a key contributing factor to systemic inflammation [82]. Simultaneously, it was observed that bacteria with anti-inflammatory potential like *Faecalibacterium prausnitzii* [83] and *Bifidobacterium adolescentis* [84], which reduce inflammation in colitis models are increased in HC individuals. We also observed that *Lactobacillus*, which possess anti-inflammatory and pathogen exclusion property [85] is increased in HC individuals. Thus, in FK subjects, the decrease in gut bacteria with anti-inflammatory properties may contribute or exacerbate the inflammatory reaction in Keratitis. The mechanism, by which these gut bacteria take part in anti-inflammatory response is probably by their ability to promote differentiation of peripheral regulatory T cells, that are primarily involved in immuno-modulation [86]. In a recent study in mice, it was demonstrated that signals from the gut microbiota activate retina specific T cells, which are involved in autoimmune Uveitis [18]. In yet another study, Andriessen et al., [20] demonstrated in a mouse model of laser-induced choroidal neovascularization that high-fat diet renders mice obese and simultaneously cause dysbiosis in the gut. Dysbiosis then drives retinal inflammation and pathological angiogenesis, thus establishing a link between dysbiosis and angiogenesis in the eye. Further, the capability of SCFAs like acetate to enter the systemic circulation increases the chance to directly exhibit their effect anywhere in the body [87].

The differences between the gut microbiomes of HC and FK subjects were also assessed by inferring the functional potential of the microbiomes (S15 and S16 Tables) from the respective taxonomic profiles. This is inferred data but decrease in beneficial and healthy microbes (such as those involved in the biosynthesis of essential vitamins (pantothenate), cofactors (Coenzyme A) and aromatic amino acids) and enrichment of several biosynthetic pathways of lipid metabolism in FK microbiomes as opposed to HC implies an imbalance in their energy metabolism [71]. Apart from this, an enormous increase in processes related to inflammation like endocytosis [88] was also observed in FK subjects (Table 4). Thus KEGG functional pathways differentiate the HC and FK microbiomes and the PCoA plot based on KEGG modules also showed FK and HC microbiomes are distinctly different (Fig 9).

To further investigate and validate the dissimilarity between gut microbiomes of FK and HC subjects, we used RF Classifier, a machine learning tool (Fig 10) used for identifying discriminating features (e.g. abundance of microbial groups across samples) from data and subsequently using the same for classification exercises (e.g. diagnosing a disease). Using this approach, highly significant variations were observed between the two groups as evident from high AUC and MCC values obtained in the ROC plot of the testing set. Thus, it appears that dysbiosis in the gut of FK subjects is strongly associated to the disease phenotype. As of now this technology is mostly practiced in research lab settings and under optimum conditions, the total time from sample collection to diagnosis should not take more than a day. On a different note, many recent studies have proposed the possibility of microbiome based diagnosis and assessment of predisposition to different medical conditions [89].

Attempts were also made to ascertain the interaction between the fungal and bacterial communities in both the HC and FK subjects to understand how these communities probably regulate one another. In HC patients (Fig 11), the fungal hub genera *Ajellomyces*, *Retroconis* and *Alternaria* include species like *Ajellomyces capsulatus*, *Ajellomyces dermatitidis* and some species of *Alternaria* that are pathogenic to either plants or human beings [90–92]. But these fungal hub genera do not discriminate between HC and FK subjects and thus their presence in the gut is intriguing.

The bacterial hubs in HC are represented by the genera *Akkermansia*, *Megamonas*, *Devosia*, *Butyricicoccus and Coprobacillus*. These five genera are common inhabitants of the human gut [93, 94] and are beneficial in several ways. For instance *Megamonas* and *Butyricicoccus* species act as probiotics [95], *Akkermansia muciniphila*, protects against metabolic disorders, including metabolic endotoxemia, adipose tissue inflammation, and insulin resistance [96] and species of the genus *Coprobacillus* confer resistance against *Clostridium difficile* colonization [97]. All the bacterial hubs had a positive interaction with several fungi and probably regulate the fungus by producing extracellular substances like SCFA [98, 99]. In the present study, the butyrate producing genera *Porphyromonas*, *Odoribacter and Acidaminococcus* exhibit negative correlation with the human pathogenic yeast genera namely *Candida* and *Meyerozyma*. In the HC gut, it appears that the organisms interact to sustain and maintain a healthy gut ecosystem.

In contrast to the HC group, the FK group showed multiple small disjoint networks (Fig 12) consisting of two fungal hub genera *Malassezia* and *Gibberella* and two bacterial hub genera *Selenomonas* and *Halomonas* (along with *Shewanella*). Both the fungal hub genera had negative interaction with other bacterial or fungal genera. Species of the genus *Malassezia* are a dominant member of human skin flora [100] and enhance gut inflammation in IBD patients [101] and are also involved in mast cells mediated inflammation [102]. *Malassezia* negatively affects the bacteria of the genera *Devosia* and *Bacillus*, which are common in the gut, *Erythrobacter* which is a anoxygenic phototroph and *Pseudoalteromonas* species, which produce anti-bacterial products. *Gibberella* is yet another hub genera in FK patients and species of this genus are associated with mucosal inflammation in Crohn’s disease [103].

In FK group, the bacterial hub genera *Selenomonas* and *Halomonas* along with *Shewanella*, positively interact with other bacteria and fungi. Species of *Selenomonas* and *Halomonas* were found to be associated with rectal and distal cancers [104] and species of *Halomonas* with obesity [105] and alcoholic cirrhosis [106]. In the present data, *Selenomonas* positively interacts with several fungi such as *Cladorhhinium foecundissimum*, a potential biocontrol fungus which controls the growth of the plant pathogen *Rhizoctonia solani* [107] and *Gymnopus dryophilus* a saprophyte, which contains anti-inflammatory beta-glucans [108]. *Halomonas* node influences positively *Cryptococcus species* which are not generally harmful to humans, except *Cryptococcus laurentii* and *Cryptococcus albidus*, which cause meningitis in patients with compromised immunity [109], *Aureobasidium pullulans* which is used in biological control of plant diseases [110] and *Glomus* species, which are mycorrhizal inoculant for agricultural soils [111]. For several other fungi, the functions are not known. To the best of our knowledge, this is the first study attempting to understand the interaction between the fungal and bacterial microbiomes in the human gut. Though the data was inferred, it does provide an insight into the positive and negative interactions operating simultaneously in the gut.

## Conclusions

Our study demonstrated dysbiosis in the gut bacterial microbiomes of FK patients compared to HC subjects. The decreased abundance of beneficial bacteria and increased abundance of pro-inflammatory and pathogenic bacteria in FK subjects may contribute to the diseased phenotype. Functional studies with specific bacteria enriched / depleted in gut microbiome of FK patients would identify the role of these bacteria or their products in disease pathogenesis, which would help to develop novel therapeutics towards sight-threatening fungal Keratitis.

## Supporting information

S1 Table Details of healthy controls and fungal Keratitis patients. (DOC)

S2 Table Relative abundances of fungal OTUs for 62 fungal microbiomes. Sparse OTUs (with < 0.001% reads) were removed.
(XLSX)

S3 Table Details of sequencing reads for 62 fungal microbiomes. (XLSX)

S4 Table Relative abundances of fungal phyla in the studied cohort. (XLSX)

S5 Table Core OTUs of the fungal microbiome libraries (OTUs having ≥ 0.001% abundance in a sample and ubiquitously present in over 80% of the samples). (DOC)

S6 Table Results of Wilcoxon test to identify discriminating fungal taxa. [results depicted at genera level; genera having median abundance > 0% in at least one class of samples (either FK or HC) included in table].
(DOC)

S7 Table Relative abundances of bacterial OTUs for 58 bacterial microbiome libraries. Sparse OTUs (with < 0.001% reads) were removed.
(XLSX)

S8 Table Details of sequencing reads for 63 bacterial microbiomes. (XLSX)

S9 Table Relative abundances of bacterial phyla in the studied cohort. Only those phyla having mean abundance > 1% are included in the table.
(XLSX)

S10 Table Relative abundances of bacterial families in the studied cohort. Only those families having mean abundance > 1% are included in the table.
(XLSX)

S11 Table Core OTUs in the bacterial microbiome libraries of both HC and FK samples. (OTUs having ≥ 0.01% abundance in a sample and ubiquitously present in over 80% of the FK and HC samples).
(DOC)

S12 Table Core OTUs (having ≥ 0.01% abundance in a sample and ubiquitously present in over 80% of the HC fecal samples) in the bacterial microbiome libraries of HC samples. (DOCX)

S13 Table Core OTUs (having ≥ 0.01% abundance in a sample and ubiquitously present in over 80% of the FK fecal samples) in the bacterial microbiome libraries of FK samples. (DOC)

S14 Table Discriminating OTUs identified through pairwise Wilcoxon test between three groups of samples (Healthy Controls (HC) vs. Fungal Keratitis_Treated (FK_T), HC vs. Fungal Keratitis_UnTreated (FK_UT) and FK_T vs. FK_UT). (DOC)

S15 Table Relative abundances of KEGG pathways inferred from respective 16S taxonomic profiles of the studied cohort. (XLSX)

S16 Table Relative abundances of KEGG modules inferred from respective 16S taxonomic profiles of the studied cohort. (XLSX)

S17 Table Summary of KEGG pathways observed in the studied cohort. The pathways that showed significantly different enrichment in each dataset were identified using Wilcoxon test (BH corrected P-value < 0.05 was considered as significant).
(XLSX)

S18 Table Summary of KEGG functional modules observed in the studied cohort. The modules that showed significantly different enrichment in each dataset were identified using Wilcoxon test (BH corrected P-value < 0.05 was considered as significant).
(XLSX)

S1 Fig Rarefaction curves of the fungal microbiome libraries of the 62 fecal samples from healthy controls and fungal Keratitis patients. (TIF)

S2 Fig Rarefaction curves of the bacterial microbiome libraries of the 58 fecal samples from healthy controls and fungal Keratitis patients. (TIF)

S3 Fig Taxonomic abundances of different bacterial families, across HC and FK samples. Only those families with > 1% mean abundance are depicted in the plot.
(TIF)
