# Correction: Alterations in the gut bacterial microbiome in fungal Keratitis patients

**DOI:** 10.1371/journal.pone.0211757

**Published:** 2019-01-30

**Authors:** Sama Kalyana Chakravarthy, Rajagopalaboopathi Jayasudha, Konduri Ranjith, Anirban Dutta, Nishal Kumar Pinna, Sharmila S. Mande, Savitri Sharma, Prashant Garg, Somasheila I. Murthy, Sisinthy Shivaji

The data published in the *PLOS ONE* article [1] are part of a larger study involving the monitoring of variations in the gut microbiome in individuals with bacterial Keratitis, fungal Keratitis and Uveitis individuals compared to healthy individuals (controls). In this larger study the healthy controls were common to the bacterial Keratitis, fungal Keratitis and Uveitis cohorts.

The microbiome data of a subset of the same healthy controls (HC005, HC009, HC012, HC013, HC014, HC016, HC017, HC018, HC022, HC025, HC026, HC028, HC029) were published in an earlier paper in the *Indian Journal of Microbiology* [2].

The Data Availability statement for this paper is incorrect. The correct statement is: Metagenomic sequencing reads can be accessed from National Center for Biotechnology Information (NCBI) BioProject accession ID PRJNA511934. All other relevant data are within the manuscript and Supporting Information files.
