# Supplementary material for: Alterations in the gut bacterial microbiome in fungal Keratitis patients
Source: PLoS One. 2018 Jun 22;13(6):e0199640. doi: 10.1371/journal.pone.0199640 (PMC6014669; doi:10.1371/journal.pone.0199640)
Supplement: S1 Table — (DOC) [file pone.0199640.s001.doc]

**S1 Table. Details of healthy controls and fungal Keratitis patients**

| **Sample ID^¥^** | **State** | **Diabetes** | **Fungi identified from corneal scraping** | **Oral antibiotics** | **Duration of antibiotic treatment** |
| --- | --- | --- | --- | --- | --- |
| FK003 | Andhra Pradesh | No | *Curvularia lunata** | No | 0 |
| FK004 | Telangana | No | Septate fungal filaments unidentified | Yes^b^ | 3 |
| FK006 | Andhra Pradesh | No | Sparsely septate fungal filaments unidentified | No | 0 |
| FK010 | Andhra Pradesh | No | Unidentified dematiaceous fungus* | No | 0 |
| FK011 | Telangana | No | *Aspergillus flavus** | No | 0 |
| FK012 | Andhra Pradesh | No | Septate fungal filaments unidentified | Yes^c^ | 15 |
| FK013 | Andhra Pradesh | No | Aseptate fungal fragments unidentified | Yes^a^ | 3 |
| FK015 | Telangana | Yes | Sparsely septate fungal filaments unidentified | Yes^a^ | 11 |
| FK018 | Chhattisgarh | No | Aseptate fungal fragments unidentified | Yes^d^ | 6 |
| FK024 | Telangana | No | *Scedosporium prolificans** | No | 0 |
| FK025 | Telangana | No | Septate fungal filaments unidentified | No | 0 |
| FK026 | Telangana | No | *Aspergillus flavus** | Yes^a^ | 30 |
| FK030 | Telangana | Yes | *Aspergillus flavus** | Yes^e^ | 24 |
| FK031 | Telangana | No | *Fusarium solani** | Yes^a^ | 3 |
| FK034 | Telangana | No | *Fusarium* species* | No | 0 |
| FK036 | Telangana | No | *Curvularia lunata** | No | 0 |
| FK039 | Andhra Pradesh | No | *Fusarium* species* | Yes^a^ | 14 |
| FK040 | Telangana | No | *Fusarium* species* | Yes^a^ | 10 |
| FK043 | Telangana | No | Aseptate fungal fragments unidentified | Yes^a^ | 5 |
| FK044 | Telangana | No | *Macrophomina phaseolina** | Yes^a^ | 1 |
| FK048 | Bihar | No | *Fusarium solani** | No | 0 |
| FK050 | Telangana | No | *Aspergillus flavus** | Yes^a^ | 7 |
| FK051 | Telangana | No | *Fusarium solani** | Yes^a^ | 12 |
| FK053 | Andhra Pradesh | No | *Aspergillus flavus** | No | 0 |
| FK055 | Andhra Pradesh | No | *Fusarium* species* | Yes^a^ | 1 |
| FK056 | Telangana | No | Septate fungal filaments unidentified | Yes^a^ | 8 |
| FK058 | Telangana | No | Septate fungal filaments unidentified | Yes^a^ | 1 |
| **Sample ID^¥^** | **State** | **Diabetes** | **Fungi identified from corneal scraping** | **Oral antibiotics** | **Duration of antibiotic treatment** |
| FK059 | Andhra Pradesh | No | Septate fungal filaments unidentified | Yes^a^ | 3 |
| FK061 | Telangana | No | Septate fungal filaments unidentified | Yes^a^ | 1 |
| FK065 | Telangana | No | *Aspergillus flavus** | Yes^c^ | 18 |
| FK066 | Andhra Pradesh | No | *Fusarium solani** | Yes^a^ | 3 |
| FK076 | Telangana | No | Septate fungal filaments unidentified | Yes^a^ | 7 |
| HC001 | Telangana | No | Not Applicable | No | Nil |
| HC002 | Telangana | No | Not Applicable | No | Nil |
| HC003 | Telangana | No | Not Applicable | No | Nil |
| HC005 | Telangana | No | Not Applicable | No | Nil |
| HC006 | Telangana | No | Not Applicable | No | Nil |
| HC007 | Andhra Pradesh | No | Not Applicable | No | Nil |
| HC009 | Telangana | No | Not Applicable | No | Nil |
| HC010 | Telangana | No | Not Applicable | No | Nil |
| HC011 | Telangana | Yes | Not Applicable | No | Nil |
| HC012 | Telangana | No | Not Applicable | No | Nil |
| HC013 | Telangana | No | Not Applicable | No | Nil |
| HC014 | Telangana | No | Not Applicable | No | Nil |
| HC015 | Andhra Pradesh | No | Not Applicable | No | Nil |
| HC016 | Andhra Pradesh | No | Not Applicable | No | Nil |
| HC017 | Telangana | No | Not Applicable | No | Nil |
| HC018 | Andhra Pradesh | No | Not Applicable | No | Nil |
| HC019 | Andhra Pradesh | No | Not Applicable | No | Nil |
| HC020 | Telangana | Yes | Not Applicable | No | Nil |
| HC021 | Telangana | No | Not Applicable | No | Nil |
| HC022 | Telangana | No | Not Applicable | No | Nil |
| HC024 | Telangana | No | Not Applicable | No | Nil |
| HC025 | Telangana | No | Not Applicable | No | Nil |
| HC026 | Telangana | No | Not Applicable | No | Nil |
| HC027 | Andhra Pradesh | Yes | Not Applicable | No | Nil |
| HC028 | Telangana | No | Not Applicable | No | Nil |
| HC029 | Telangana | No | Not Applicable | No | Nil |
| HC031 | Maharashtra | No | Not Applicable | No | Nil |
| HC033 | Andhra Pradesh | No | Not Applicable | No | Nil |
| HC034 | Andhra Pradesh | No | Not Applicable | No | Nil |
| HC035 | Telangana | No | Not Applicable | No | Nil |
| HC036 | Telangana | No | Not Applicable | No | Nil |

* Identified through colony characteristics and microscopic features

^a^ Ketokonazole 200 mg - 2 times / day

^b^ Ofloxacin 400 mg - 1 time / day, fluconazole 150 mg - 2 times / day

^c^ Itraconazole 100 mg - 2 times / day

^d^ Ketokonazole 200 mg - 2 times / day for 5 days followed by itraconazole 100 mg - 2 times / day

for one day

^e^ Ketokonazole 200 mg - 2 times / day for 20 days followed by itraconazole 100 mg - 2 times /

day for 4 days

^¥^ The healthy controls (HC, n=31) and fungal Keratitis (FK, n=32) patients were matched for age (P = 0.188) and sex (males [P = 0.312]; females [P = 0.426]).
