# Supplementary material for: Alterations in the gut bacterial microbiome in fungal Keratitis patients
Source: PLoS One. 2018 Jun 22;13(6):e0199640. doi: 10.1371/journal.pone.0199640 (PMC6014669; doi:10.1371/journal.pone.0199640)
Supplement: S5 Table — (DOC) [file pone.0199640.s005.doc]

**S5 Table. Core OTUs of the fungal microbiome libraries (OTUs having ≥ 0.001% abundance in a sample and ubiquitously present in over 80% of the samples)**

| **Taxonomic Affiliation (OTU Level) of Core OTUs** | **Number of OTUs** |
| --- | --- |
| **Common core OTUs in both HC and FK samples** | 0 |
| **Core OTUs in FK samples** |  |
| k__Fungi;p__Ascomycota;c__Saccharomycetes;o__Saccharomycetales;f__Incertae_sedis;g__*Candida*;s__*Candida*_*albicans*;OTU_GQ280299 | 1 |
| k__Fungi;p__Ascomycota;c__Saccharomycetes;o__Saccharomycetales;f__Incertae_sedis;g__*Candida*;s__*Candida*_*tropicalis*;OTU_EU288196 | 1 |
| **Core OTUs in HC samples** | 0 |
