# Supplementary material for: Alterations in the gut bacterial microbiome in fungal Keratitis patients
Source: PLoS One. 2018 Jun 22;13(6):e0199640. doi: 10.1371/journal.pone.0199640 (PMC6014669; doi:10.1371/journal.pone.0199640)
Supplement: S6 Table — [results depicted at genera level; genera having median abundance > 0% in at least one class of samples (either FK or HC) included in table]. (DOC) [file pone.0199640.s006.doc]

**S6 Table. Results of Wilcoxon test to identify discriminating fungal taxa** [results depicted at genera level; genera having median abundance > 0% in at least one class of samples (either FK or HC) included in table]

| **Genus** | **Median abundance (%)** | | **Wilcoxon test -P value (BH - corrected)** |
| --- | --- | --- | --- |
|  | **FK** | **HC** |  |
| *Candida* | 1.1757 | 0.4072 | 0.829 |
| *Malassezia* | 0.1658 | 0.0655 | 0.831 |
| *Fusarium* | 0.1038 | 0.0287 | 0.831 |
| *Cladosporium* | 0.0617 | 0.0814 | 0.831 |
| *Mortierella* | 0.0660 | 0.0485 | 1.000 |
| *Aspergillus* | 0.0405 | 0.0111 | 0.636 |
| *Saccharomyces* | 0.0118 | 0.0312 | 0.977 |
| *Pichia* | 0.0202 | 0.0168 | 0.966 |
| *Kluyveromyces* | 0.0004 | 0.0173 | 0.139 |
| *Eurotium* | 0.0138 | 0.0026 | 0.520 |
| *Alternaria* | 0.0022 | 0.0122 | 0.753 |
| *Rhizopus* | 0.0030 | 0.0100 | 1.000 |
| *Lasiodiplodia* | 0.0021 | 0.0003 | 0.974 |
| *Trichosporon* | 0.0014 | 0.0001 | 0.831 |
| *Clavispora* | 0.0010 | 0.0012 | 0.977 |
| *Emericella* | 0.0001 | 0.0005 | 0.966 |
| *Trichocladium* | 0.0003 | 0.0001 | 0.977 |
| *Coprinellus* | 0.0000 | 0.0002 | 1.000 |
| *Meyerozyma* | 0.0002 | 0.0000 | 0.677 |
| *Cochliobolus* | 0.0002 | 0.0002 | 0.965 |
