# Supplementary material for: Alterations in the gut bacterial microbiome in fungal Keratitis patients
Source: PLoS One. 2018 Jun 22;13(6):e0199640. doi: 10.1371/journal.pone.0199640 (PMC6014669; doi:10.1371/journal.pone.0199640)
Supplement: S11 Table — (OTUs having ≥ 0.01% abundance in a sample and ubiquitously present in over 80% of the FK and HC samples). (DOC) [file pone.0199640.s011.doc]

**S11 Table. Core OTUs in the bacterial microbiome libraries of both HC and FK samples (OTUs having ≥ 0.01% abundance in a sample and ubiquitously present in over 80% of the FK and HC samples)**

| **Lineage** | **Number of OTUs** | **OTU ID** |
| --- | --- | --- |
| **Core OTUs in both HC and FK samples assigned at order level:** | | |
| p__Firmicutes; c__Clostridia; o__Clostridiales | 2 | 353214, 366584 |
| **Core OTUs in both HC and FK samples assigned at family level:** | | |
| p__Actinobacteria; c__Coriobacteriia; o__Coriobacteriales; f__Coriobacteriaceae | 1 | 183104 |
| p__Bacteroidetes; c__Bacteroidia; o__Bacteroidales; f__S24-7 | 1 | 844589 |
| p__Firmicutes; c__Clostridia; o__Clostridiales; f__Lachnospiraceae | 11 | 216111, 358104, 368261, 369027, 509383, 526468, 546876, 562038, 574689, 708680, 791522 |
| p__Firmicutes; c__Clostridia; o__Clostridiales; f__Ruminococcaceae | 8 | 1062061, 361811, 362078, 367213, 370086, 551902, 560535, 819181 |
| p__Firmicutes; c__Erysipelotrichi; o__Erysipelotrichales; f__Erysipelotrichaceae | 1 | 225636 |
| p__Proteobacteria; c__Gammaproteobacteria; o__Enterobacteriales; f__Enterobacteriaceae | 2 | 782953, 797229 |
| **Core OTUs in both HC and FK samples assigned at genera level:** | | |
| p__Actinobacteria; c__Actinobacteria; o__Bifidobacteriales; f__Bifidobacteriaceae; g__*Bifidobacterium* | 1 | 553611 |
| p__Actinobacteria; c__Coriobacteriia; o__Coriobacteriales; f__Coriobacteriaceae; g__*Slackia* | 1 | 367139 |
| p__Bacteroidetes; c__Bacteroidia; o__Bacteroidales; f__[Paraprevotellaceae]; g__[*Prevotella*] | 1 | denovo_19847 |
| p__Bacteroidetes; c__Bacteroidia; o__Bacteroidales; f__Bacteroidaceae; g__*Bacteroides* | 3 | 183480, 577170, 589277 |
| p__Firmicutes; c__Clostridia; o__Clostridiales; f__Clostridiaceae; g__*SMB53* | 1 | 712677 |
| p__Firmicutes; c__Clostridia; o__Clostridiales; f__Lachnospiraceae; g__*Blautia* | 1 | 570507 |
| p__Firmicutes; c__Clostridia; o__Clostridiales; f__Lachnospiraceae; g__*Coprococcus* | 1 | 804526 |
| p__Firmicutes; c__Clostridia; o__Clostridiales; f__Lachnospiraceae; g__*Lachnospira* | 3 | 309433, 369486, 370098 |
| p__Firmicutes; c__Clostridia; o__Clostridiales; f__Lachnospiraceae; g__*Roseburia* | 1 | 531436 |
| **Lineage** | **Number of OTUs** | **OTU ID** |
| p__Firmicutes; c__Clostridia; o__Clostridiales; f__Ruminococcaceae; g__*Oscillospira* | 2 | 304777, 314582 |
| p__Firmicutes; c__Clostridia; o__Clostridiales; f__Ruminococcaceae; g__*Ruminococcus* | 2 | 365965, 523140 |
| p__Firmicutes; c__Clostridia; o__Clostridiales; f__Veillonellaceae; g__*Dialister* | 1 | 264552 |
| p__Firmicutes; c__Clostridia; o__Clostridiales; f__Veillonellaceae; g__*Megamonas* | 1 | 325808 |
| p__Firmicutes; c__Clostridia; o__Clostridiales; f__Veillonellaceae; g__*Megasphaera* | 1 | 264967 |
| p__Firmicutes; c__Erysipelotrichi; o__Erysipelotrichales; f__Erysipelotrichaceae; g__*Catenibacterium* | 1 | 330294 |
| p__Proteobacteria; c__Betaproteobacteria; o__Burkholderiales; f__Alcaligenaceae; g__*Sutterella* | 1 | 1820513 |
| p__Proteobacteria; c__Deltaproteobacteria; o__Desulfovibrionales; f__Desulfovibrionaceae; g__*Bilophila* | 1 | 841907 |
| p__Proteobacteria; c__Gammaproteobacteria; o__Aeromonadales; f__Succinivibrionaceae; g__*Succinivibrio* | 1 | 516159 |
| **Core OTUs in both HC and FK samples assigned at species level:** | | |
| p__Actinobacteria; c__Actinobacteria; o__Bifidobacteriales; f__Bifidobacteriaceae; g__*Bifidobacterium*; s__*adolescentis* | 1 | 584375 |
| p__Actinobacteria; c__Actinobacteria; o__Bifidobacteriales; f__Bifidobacteriaceae; g__*Bifidobacterium*; s__*longum* | 1 | 559527 |
| p__Actinobacteria; c__Coriobacteriia; o__Coriobacteriales; f__Coriobacteriaceae; g__*Collinsella*; s__*aerofaciens* | 1 | 368175 |
| p__Bacteroidetes; c__Bacteroidia; o__Bacteroidales; f__Bacteroidaceae; g__*Bacteroides*; s__*ovatus* | 1 | 535375 |
| p__Bacteroidetes; c__Bacteroidia; o__Bacteroidales; f__Bacteroidaceae; g__*Bacteroides*; s__*plebeius* | 1 | 365496 |
| p__Bacteroidetes; c__Bacteroidia; o__Bacteroidales; f__Bacteroidaceae; g__*Bacteroides*; s__*uniformis* | 1 | 589071 |
| p__Bacteroidetes; c__Bacteroidia; o__Bacteroidales; f__Porphyromonadaceae; g__*Parabacteroides*; s__*distasonis* | 1 | 585914 |
| p__Bacteroidetes; c__Bacteroidia; o__Bacteroidales; f__Prevotellaceae; g__*Prevotella*; s__*copri* | 3 | 568118, 588929, 589329 |
| p__Firmicutes; c__Bacilli; o__Lactobacillales; f__Lactobacillaceae; g__*Lactobacillus*; s__*ruminis* | 1 | denovo_11856 |
| p__Firmicutes; c__Clostridia; o__Clostridiales; f__Lachnospiraceae; g__*Dorea*; s__*formicigenerans* | 1 | 1076587 |
| p__Firmicutes; c__Clostridia; o__Clostridiales; f__Ruminococcaceae; g__*Faecalibacterium*; s__*prausnitzii* | 3 | 525215, 525698, 851865 |
| p__Firmicutes; c__Clostridia; o__Clostridiales; f__Veillonellaceae; g__*Mitsuokella*; s__*multacida* | 1 | 306124 |
| **Total** | **66** |  |
