# Supplementary material for: Alterations in the gut bacterial microbiome in fungal Keratitis patients
Source: PLoS One. 2018 Jun 22;13(6):e0199640. doi: 10.1371/journal.pone.0199640 (PMC6014669; doi:10.1371/journal.pone.0199640)
Supplement: S12 Table — (DOCX) [file pone.0199640.s012.docx]

**S12 Table. Core OTUs (having ≥ 0.01% abundance in a sample and ubiquitously present in over 80% of the HC fecal samples) in the bacterial microbiome libraries of HC samples**

| **Lineage** | **Number of OTUs** | **OTU ID** |
| --- | --- | --- |
| **HC core OTU assigned at order level:** | | |
| p__Firmicutes; c__Clostridia; o__Clostridiales | 3 | 361727, 369429, 4429981 |
| **HC core OTUs assigned at family level:** | | |
| p__Bacteroidetes; c__Bacteroidia; o__Bacteroidales; f__[Barnesiellaceae] | 1 | 315846 |
| p__Firmicutes; c__Clostridia; o__Clostridiales; f__Clostridiaceae | 2 | 337379, 843459 |
| p__Firmicutes; c__Clostridia; o__Clostridiales; f__Lachnospiraceae | 6 | 211935, 291493, 338992, 352733, 360329, 363400 |
| p__Firmicutes; c__Clostridia; o__Clostridiales; f__Ruminococcaceae | 8 | 191332, 228061, 349680, 355685, 359175, 369109, 539328, 580521 |
| p__Proteobacteria; c__Gammaproteobacteria; o__Enterobacteriales; f__Enterobacteriaceae | 2 | 813457, 821080 |
| **HC Core OTUs assigned at genera level:** | | |
| p__Bacteroidetes; c__Bacteroidia; o__Bacteroidales; f__Bacteroidaceae; g__*Bacteroides* | 4 | 339013, 351292, 364903, 364926 |
| p__Bacteroidetes; c__Bacteroidia; o__Bacteroidales; f__Porphyromonadaceae; g__*Parabacteroides* | 2 | 198866, 851323 |
| p__Firmicutes; c__Bacilli; o__Lactobacillales; f__Streptococcaceae; g__*Streptococcus* | 2 | 1082539, 1085832 |
| p__Firmicutes; c__Clostridia; o__Clostridiales; f__Clostridiaceae; g__*Clostridium* | 1 | 1105343 |
| p__Firmicutes; c__Clostridia; o__Clostridiales; f__Lachnospiraceae; g__*Dorea* | 3 | 1105552, 523542, 909065 |
| p__Firmicutes; c__Clostridia; o__Clostridiales; f__Lachnospiraceae; g__*Lachnospira* | 2 | 314095, 349257 |
| p__Firmicutes; c__Clostridia; o__Clostridiales; f__Ruminococcaceae; g__*Oscillospira* | 2 | 310886, 359563 |
| p__Firmicutes; c__Clostridia; o__Clostridiales; f__Ruminococcaceae; g__*Ruminococcus* | 4 | 193755, 304211, 344523, 591635 |
| p__Firmicutes; c__Clostridia; o__Clostridiales; f__Veillonellaceae; g__*Dialister* | 1 | 583746 |
| p__Firmicutes; c__Clostridia; o__Clostridiales; f__Veillonellaceae; g__*Megasphaera* | 2 | 266210, 817140 |
| p__Proteobacteria; c__Betaproteobacteria; o__Burkholderiales; f__Alcaligenaceae; g__*Sutterella* | 2 | 173726, 359809 |
| p__Proteobacteria; c__Gammaproteobacteria; o__Enterobacteriales; f__Enterobacteriaceae; g__*Klebsiella* | 1 | 813217 |
| **Lineage** | **Number of OTUs** | **OTU ID** |
| **HC Core OTUs assigned at species level:** | | |
| p__Bacteroidetes; c__Bacteroidia; o__Bacteroidales; f__Prevotellaceae; g__*Prevotella*; s__*copri* | 2 | 307571, 345899 |
| p__Bacteroidetes; c__Bacteroidia; o__Bacteroidales; f__Prevotellaceae; g__*Prevotella*; s__*stercorea* | 1 | 524371 |
| p__Firmicutes; c__Clostridia; o__Clostridiales; f__Ruminococcaceae; g__*Faecalibacterium*; s__*prausnitzii* | 1 | 367433 |
| p__Firmicutes; c__Clostridia; o__Clostridiales; f__Veillonellaceae; g__*Veillonella*; s__*dispar* | 1 | 342427 |
| p__Firmicutes; c__Erysipelotrichi; o__Erysipelotrichales; f__Erysipelotrichaceae; g__[*Eubacterium*]; s__*biforme* | 2 | 197105, 524884 |
| p__Proteobacteria; c__Gammaproteobacteria; o__Pasteurellales; f__Pasteurellaceae; g__*Haemophilus*; s__*parainfluenzae* | 1 | 968675 |
| p__Firmicutes; c__Clostridia; o__Clostridiales; f__Lachnospiraceae; g__*Coprococcus*; s__*eutactus* | 1 | 190679 |
| **Total** | **57** |  |
