# Supplementary material for: Alterations in the gut bacterial microbiome in fungal Keratitis patients
Source: PLoS One. 2018 Jun 22;13(6):e0199640. doi: 10.1371/journal.pone.0199640 (PMC6014669; doi:10.1371/journal.pone.0199640)
Supplement: S13 Table — (DOC) [file pone.0199640.s013.doc]

**S13 Table. Core OTUs (having ≥ 0.01% abundance in a sample and ubiquitously present in over 80% of the FK fecal samples) in the bacterial microbiome libraries of FK samples**

| **Lineage** | **Number of OTUs** | **OTU ID** |
| --- | --- | --- |
| **FK core OTU assigned at order level:** | | |
| p__Firmicutes; c__Clostridia; o__Clostridiales | 1 | 552380 |
| **FK core OTUs assigned at family level:** |  |  |
| p__Bacteroidetes; c__Bacteroidia; o__Bacteroidales; f__S24-7 | 1 | 577228 |
| p__Firmicutes; c__Clostridia; o__Clostridiales; f__Lachnospiraceae | 1 | 367813 |
| p__Firmicutes; c__Clostridia; o__Clostridiales; f__Ruminococcaceae | 3 | 309720, 351927, 584951 |
| p__Proteobacteria; c__Gammaproteobacteria; o__Enterobacteriales; f__Enterobacteriaceae | 1 | 1111294 |
| **FK Core OTUs assigned at genera level:** | | |
| p__Bacteroidetes; c__Bacteroidia; o__Bacteroidales; f__Bacteroidaceae; g__*Bacteroides* | 1 | 583117 |
| p__Firmicutes; c__Bacilli; o__Lactobacillales; f__Streptococcaceae; g__*Streptococcus* | 1 | 532232 |
| p__Proteobacteria; c__Deltaproteobacteria; o__Desulfovibrionales; f__Desulfovibrionaceae; g__*Desulfovibrio* | 1 | 4453773 |
| **FK Core OTUs assigned at species level:** | | |
| p__Bacteroidetes; c__Bacteroidia; o__Bacteroidales; f__Bacteroidaceae; g__*Bacteroides*; s__*fragilis* | 1 | 2200896 |
| p__Firmicutes; c__Clostridia; o__Clostridiales; f__Lachnospiraceae; g__[*Ruminococcus*]; s__*gnavus* | 1 | 360015 |
| **Total** | **12** |  |
