# Supplementary material for: Alterations in the gut bacterial microbiome in fungal Keratitis patients
Source: PLoS One. 2018 Jun 22;13(6):e0199640. doi: 10.1371/journal.pone.0199640 (PMC6014669; doi:10.1371/journal.pone.0199640)
Supplement: S14 Table — (DOC) [file pone.0199640.s014.doc]

**S14 Table. Discriminating OTUs identified through pairwise Wilcoxon test between three groups of samples (Healthy Controls (HC) vs. Fungal Keratitis_Treated (FK_T), HC vs. Fungal Keratitis_UnTreated (FK_UT) and FK_T vs. FK_UT).** The BH corrected P-values (significant at P < 0.05) are highlighted in the Table. OTUs having a minimum median abundance of > 0.01% in one of the three groups are listed

| **OTU ID** | **Taxonomic**  **affiliation** | **Median abundance (%)** | | | | | **Wilcoxon test -P value  (BH – corrected)** | | | | |
| --- | --- | --- | --- | --- | --- | --- | --- | --- | --- | --- | --- |
|  |  | **FK_UT** | **HC** | | **FK_T** | | **HC**  **vs. FK_UT** | **HC**  **vs. FK_T** | | **FK_UT vs.**  **FK_T** | |
| OTU264967 | *Megasphaera* | 0.29 | 3.98 | | 0.17 | | 0.017 | 0.001 | | 0.886 | |
| OTU708680 | [Family] Lachnospiraceae | 0.59 | 2.11 | | 0.58 | | 0.002 | 0.011 | | 0.970 | |
| OTU2200896 | *Bacteroides fragilis* | 0.48 | 0.01 | | 0.06 | | 0.003 | 0.017 | | 0.658 | |
| OTU369486 | *Lachnospira* | 0.19 | 0.43 | | 0.10 | | 0.052 | 0.000 | | 0.658 | |
| OTU306124 | *Mitsuokella multacida* | 0.10 | 0.41 | | 0.04 | | 0.014 | 0.004 | | 0.723 | |
| OTU365496 | *Bacteroides plebeius* | 0.08 | 0.35 | | 0.05 | | 0.038 | 0.003 | | 0.675 | |
| OTU817140 | *Megasphaera* | 0.01 | 0.24 | | 0.01 | | 0.007 | 0.001 | | 0.985 | |
| OTU309433 | *Lachnospira* | 0.07 | 0.23 | | 0.06 | | 0.153 | 0.003 | | 0.658 | |
| OTU183480 | *Bacteroides* | 0.05 | 0.22 | | 0.03 | | 0.047 | 0.004 | | 0.886 | |
| OTU191332 | [Family] Ruminococcaceae | 0.04 | 0.19 | | 0.03 | | 0.026 | 0.006 | | 0.861 | |
| OTU314095 | *Lachnospira* | 0.06 | 0.17 | | 0.03 | | 0.014 | 0.001 | | 0.658 | |
| OTU349257 | *Lachnospira* | 0.04 | 0.17 | | 0.02 | | 0.008 | 0.001 | | 0.769 | |
| OTU531436 | *Roseburia* | 0.05 | 0.14 | | 0.04 | | 0.002 | 0.001 | | 0.893 | |
| OTU298050 | *Megasphaera* | 0.02 | 0.14 | | 0.00 | | 0.070 | 0.002 | | 0.769 | |
| OTUdenovo11856 | *Lactobacillus ruminis* | 0.04 | 0.12 | | 0.02 | | 0.040 | 0.001 | | 0.658 | |
| OTU304211 | *Ruminococcus* | 0.01 | 0.12 | | 0.02 | | 0.008 | 0.021 | | 0.985 | |
| OTU298592 | [Family] Fusobacteriaceae | 0.09 | 0.00 | | 0.04 | | 0.004 | 0.089 | | 0.658 | |
| OTU344523 | *Ruminococcus* | 0.02 | 0.07 | | 0.01 | | 0.206 | 0.003 | | 0.658 | |
| OTU1105343 | *Clostridium* | 0.04 | 0.07 | | 0.01 | | 0.611 | 0.004 | | 0.658 | |
| OTU266210 | *Megasphaera* | 0.00 | 0.06 | | 0.01 | | 0.006 | 0.001 | | 0.985 | |
| OTU215097 | *Sutterella* | 0.01 | 0.06 | | 0.00 | | 0.070 | 0.001 | | 0.658 | |
| OTU315846 | [Family] Barnesiellaceae | 0.02 | 0.05 | | 0.00 | | 0.076 | 0.001 | | 0.723 | |
| OTU211935 | [Family] Lachnospiraceae | 0.01 | 0.05 | | 0.01 | | 0.008 | 0.003 | | 0.886 | |
| **OTU ID** | **Taxonomic**  **affiliation** | **Median abundance (%)** | | | | | **Wilcoxon test -P value  (BH – corrected)** | | | | |
|  |  | **FK_UT** | | **HC** | | **FK_T** |  | | | | |
|  |  |  |  |  |  |  | **HC**  **vs. FK_UT** | | **HC**  **vs. FK_T** | | **FK_UT vs.**  **FK_T** |
| OTU539328 | [Family] Ruminococcaceae | 0.02 | | 0.05 | | 0.01 | 0.197 | | 0.005 | | 0.658 |
| OTU339013 | *Bacteroides* | 0.03 | | 0.05 | | 0.01 | 0.575 | | 0.002 | | 0.658 |
| OTU659361 | *Dorea* | 0.04 | | 0.00 | | 0.02 | 0.007 | | 0.011 | | 0.760 |
| OTU338992 | [Family] Lachnospiraceae | 0.02 | | 0.04 | | 0.02 | 0.020 | | 0.004 | | 0.998 |
| OTU366392 | [Family] Coriobacteriaceae | 0.00 | | 0.04 | | 0.00 | 0.206 | | 0.003 | | 0.760 |
| OTU849612 | [Family] TK06 | 0.03 | | 0.00 | | 0.01 | 0.003 | | 0.020 | | 0.658 |
| OTU149335 | *Mitsuokella* | 0.00 | | 0.03 | | 0.00 | 0.011 | | 0.001 | | 0.760 |
| OTU4429981 | [Order] Clostridiales | 0.00 | | 0.02 | | 0.00 | 0.024 | | 0.004 | | 0.998 |
| OTU4332082 | *Roseburia* | 0.01 | | 0.02 | | 0.00 | 0.185 | | 0.002 | | 0.658 |
| OTU514045 | *Treponema* | 0.02 | | 0.00 | | 0.01 | 0.003 | | 0.005 | | 0.658 |
| OTU79134 | [Family] ZA3409c | 0.02 | | 0.00 | | 0.00 | 0.003 | | 0.013 | | 0.658 |
| OTU357471 | [Order] Clostridiales | 0.01 | | 0.02 | | 0.00 | 0.218 | | 0.002 | | 0.658 |
| OTU318061 | [Class] Alphaproteobacteria | 0.02 | | 0.00 | | 0.00 | 0.002 | | 0.004 | | 0.658 |
| OTU687245 | *Mitsuokella* | 0.00 | | 0.01 | | 0.00 | 0.014 | | 0.001 | | 0.658 |
| OTU816299 | [Family] Coriobacteriaceae | 0.00 | | 0.01 | | 0.00 | 0.009 | | 0.001 | | 0.915 |
| OTU780650 | [Family] Clostridiaceae | 0.01 | | 0.01 | | 0.00 | 0.484 | | 0.002 | | 0.658 |
| OTU581021 | [Family] Enterobacteriaceae | 0.00 | | 0.01 | | 0.00 | 0.007 | | 0.004 | | 0.985 |
| OTU195271 | [Family] Lachnospiraceae | 0.01 | | 0.00 | | 0.01 | 0.005 | | 0.310 | | 0.658 |
| OTU185659 | *Acidaminococcus* | 0.00 | | 0.01 | | 0.00 | 0.104 | | 0.001 | | 0.658 |
| OTU320696 | [Order] Acidimicrobiales | 0.01 | | 0.00 | | 0.00 | 0.003 | | 0.008 | | 0.658 |
| OTU820764 | [Family] Veillonellaceae | 0.00 | | 0.01 | | 0.00 | 0.008 | | 0.000 | | 0.886 |
| OTU350121 | [Family] Ruminococcaceae | 0.00 | | 0.01 | | 0.00 | 0.061 | | 0.005 | | 0.886 |
| OTU830290 | *Pseudoalteromonas* | 0.01 | | 0.00 | | 0.00 | 0.002 | | 0.003 | | 0.658 |
| OTU328892 | *Roseburia* | 0.00 | | 0.01 | | 0.00 | 0.206 | | 0.003 | | 0.658 |
| OTU639502 | [Family] OCS155 | 0.01 | | 0.00 | | 0.00 | 0.003 | | 0.007 | | 0.658 |
| OTU176306 | [Family] Lachnospiraceae | 0.00 | | 0.01 | | 0.00 | 0.005 | | 0.007 | | 0.980 |

“Fungal Keratitis_Treated” includes patients treated with either antifungal drugs (21 patients) or both antibacterial and antifungal drugs (1 patient) [See Additional file 1: Table S1].
