# Supplementary figures and images for: Alterations in the gut bacterial microbiome in fungal Keratitis patients
Source: PLoS One. 2018 Jun 22;13(6):e0199640. doi: 10.1371/journal.pone.0199640 (PMC6014669; doi:10.1371/journal.pone.0199640)

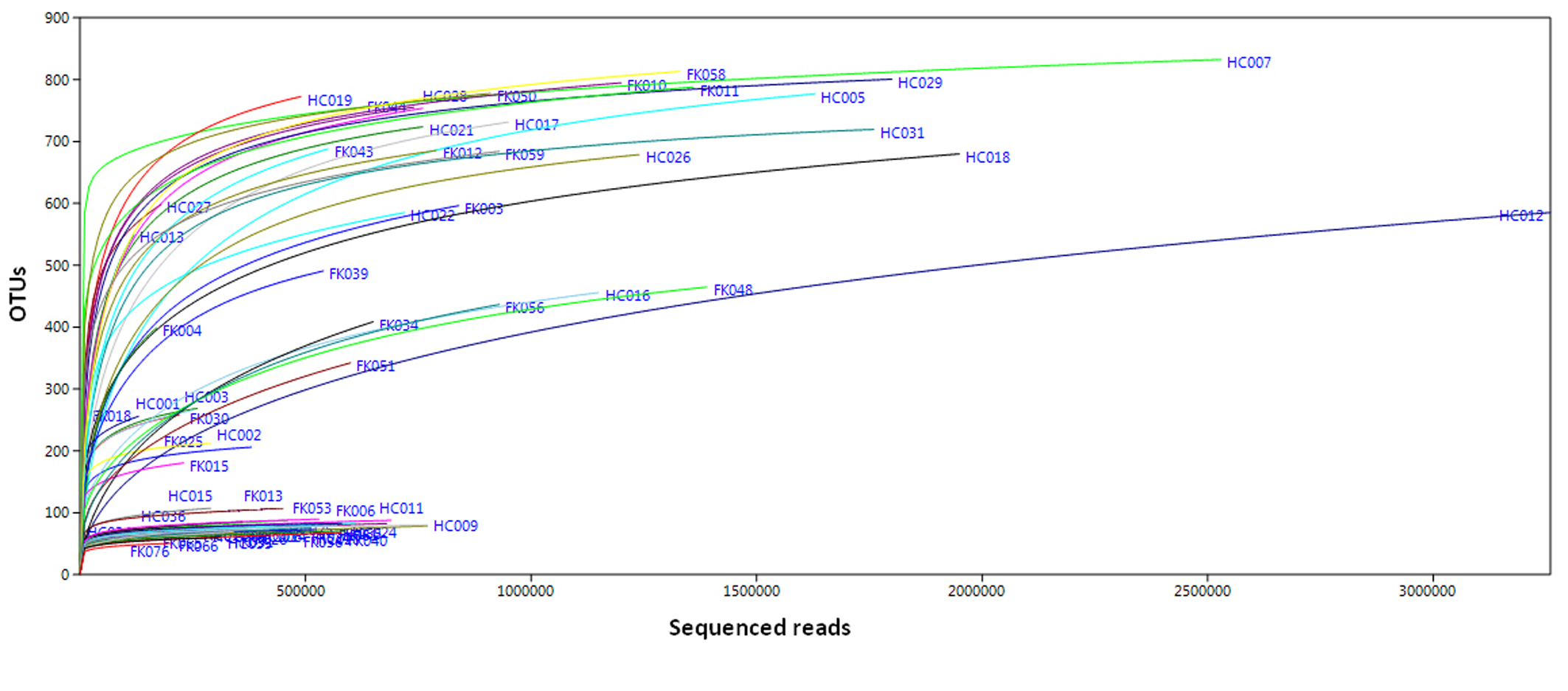

Supplement: S1 Fig — (TIF) [file pone.0199640.s019.tif]

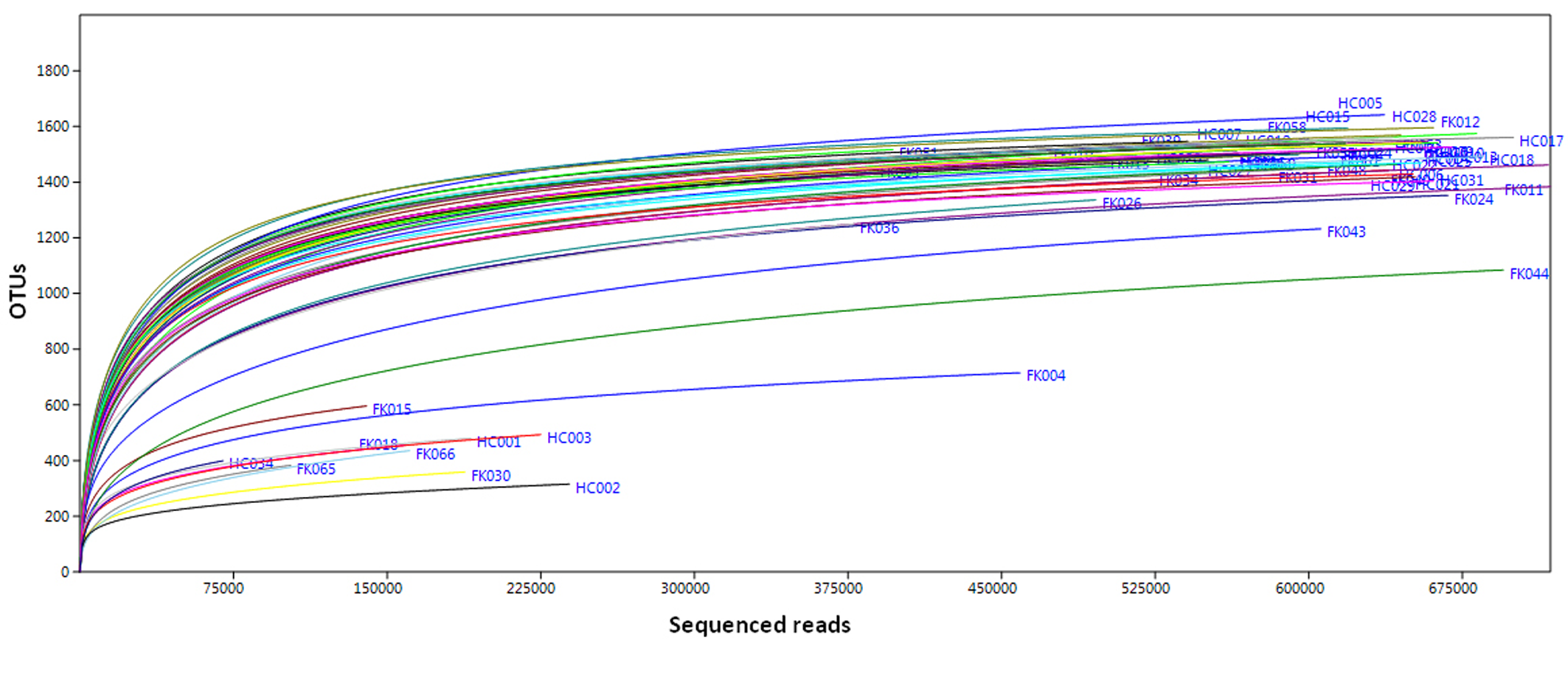

Supplement: S2 Fig — (TIF) [file pone.0199640.s020.tif]

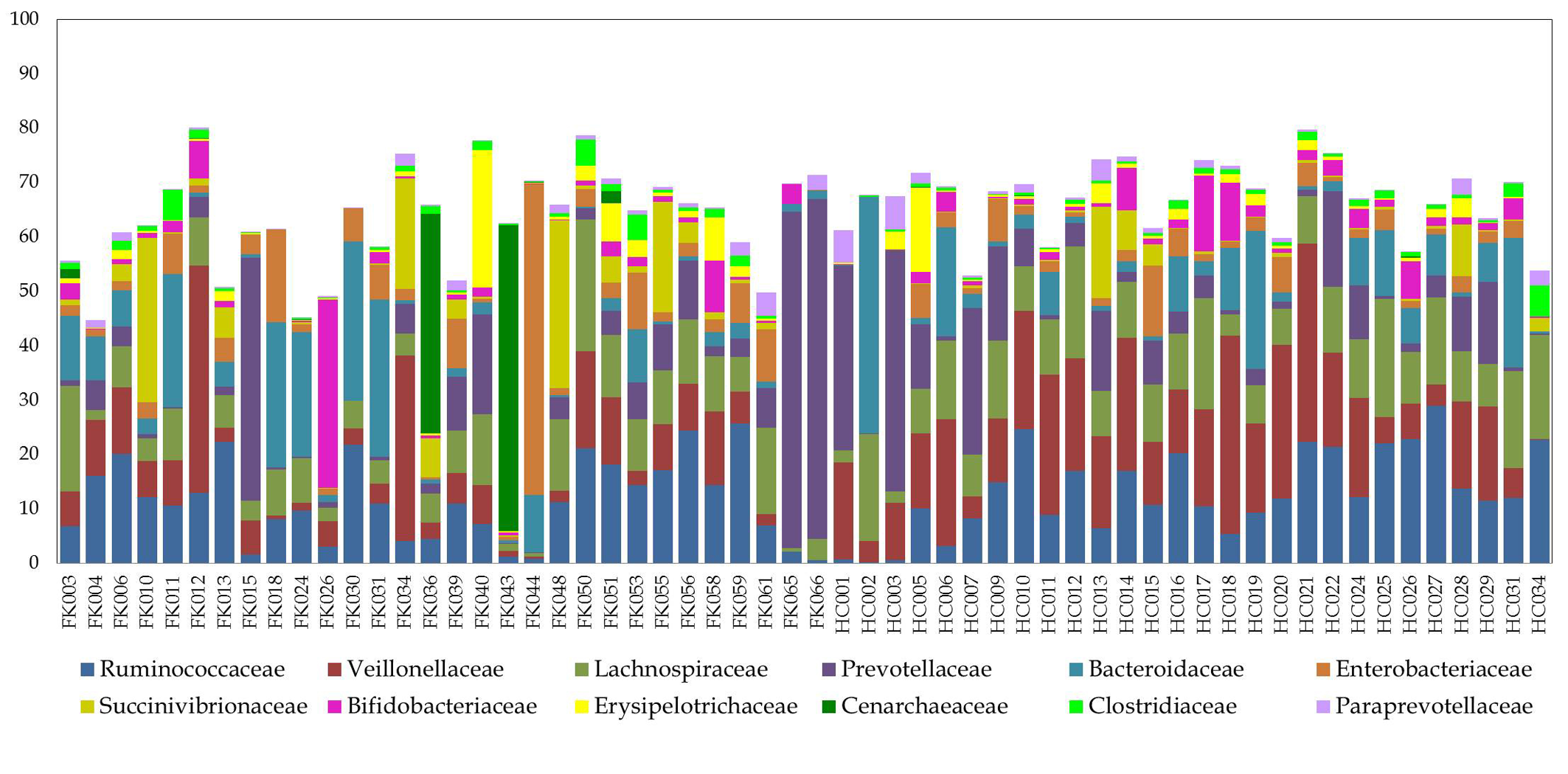

Supplement: S3 Fig — Only those families with > 1% mean abundance are depicted in the plot. (TIF) [file pone.0199640.s021.tif]
